# Nanomedicine-induced pyroptosis for anti-tumor immunotherapy: Mechanism analysis and application prospects

**DOI:** 10.1016/j.apsb.2025.05.021

**Published:** 2025-05-26

**Authors:** Yuelin Huang, Chunting Wang, Yanhong Chen, Dengbin Wang, Defan Yao

**Affiliations:** a Shanghai University of Sport, Shanghai 200438, China; b Department of Radiology, Xinhua Hospital, Shanghai Jiao Tong University School of Medicine, Shanghai 200092, China; c College of Health Science and Technology, Shanghai Jiao Tong University School of Medicine, Shanghai 200025, China

**Keywords:** Pyroptosis, Nanomedicine, Anti-tumor immunity, Cancer, Tumor microenvironments, Gasdermin protein, Reactive oxygen species, Immunogenic cell death

## Abstract

Pyroptosis is a new type of programmed cell death that can efficiently enhance the immune response by inducing cell lysis and inflammation, thereby facilitating tumor immunotherapy. Recently, an increasing number of studies have revealed close relationships between pyroptosis and nanomedicine, which has been regarded as a new strategy for developing nanomedicine-based immunotherapy for highly effective therapy of various cancers. In this review, the development and associated signaling pathways for pyroptosis, including the correlation between pyroptosis and anti-tumor immunity, were first presented. Then, various nanomedicines that induce pyroptosis for tumor therapy, especially immunotherapy, were systematically discussed. Finally, the current challenges and constructive perspectives in this field were proposed.

## Graphical abstract

Pyroptosis promotes immune activation by releasing damage-associated molecular patterns and pro-inflammatory cytokines. Nanomedicines synergize with multiple therapeutic modalities by inducing pyroptosis to further enhance the efficacy of anti-tumor immunotherapeutic strategies.

**Figure:**
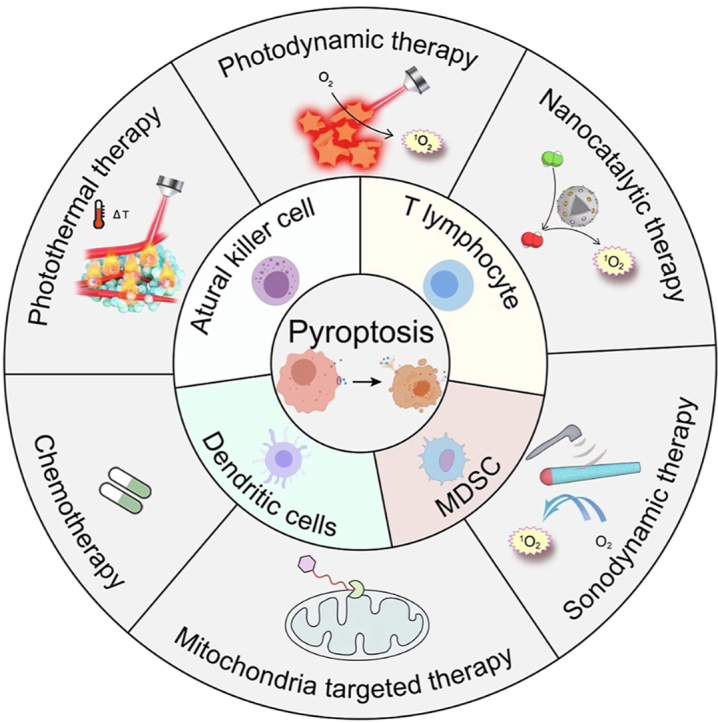

## 1 Introduction

Cancer is the leading cause of human mortality and poses a significant threat to public health. Conventional cancer treatments such as chemotherapy, radiotherapy, and surgery have limitations, including adverse effects and limited effectiveness. Solid tumors cannot be effectively treated with a single conventional immunotherapy approach. Combining immunotherapy with established treatments is being sought to improve outcomes and reduce adverse effects. Tumor immunotherapy is emerging as an important modality for treating tumor growth.

Tumor immunotherapy is an emerging therapeutic strategy aimed at fighting tumors by activating the body’s immune system. In recent years, the application of nanomedicine in the field of tumor immunotherapy has attracted more and more attention, among which nanomedicine-induced pyroptosis has a broad application prospect as a novel tumor immunotherapy method. Activation of pyroptosis by nanomedicine leads to the release of many inflammatory mediators, which modulate their immune system to exhibit anti-tumor effects. Therefore, the development of precisely functionalized nanomedicines targeting complex tumor microenvironments (TME) and specific tumors provides a novel approach to induce pyroptosis in tumor therapy.

First, nanomedicines could accurately target tumors, which can improve the therapy effect; second, nanomedicines are easy to modify, which can realize the functionalization and multifaceted application of materials. These advantages of the materials are utilized to address biosafety issues and further improve their efficacy in tumor therapy. In addition, the pyroptosis mechanism can activate molecular pathways by recognizing signals emitted by bacteria and viruses, and the upregulation of gasdermin gene expression can also lead to pyroptosis. Therefore, by developing nanomedicine to induce pyroptosis to increase their value for tumor therapy applications^1^. Additionally, nanomedicine advances have the potential to improve treatment precision and efficacy, leading to more effective tumor therapy.

**Scheme 1:**
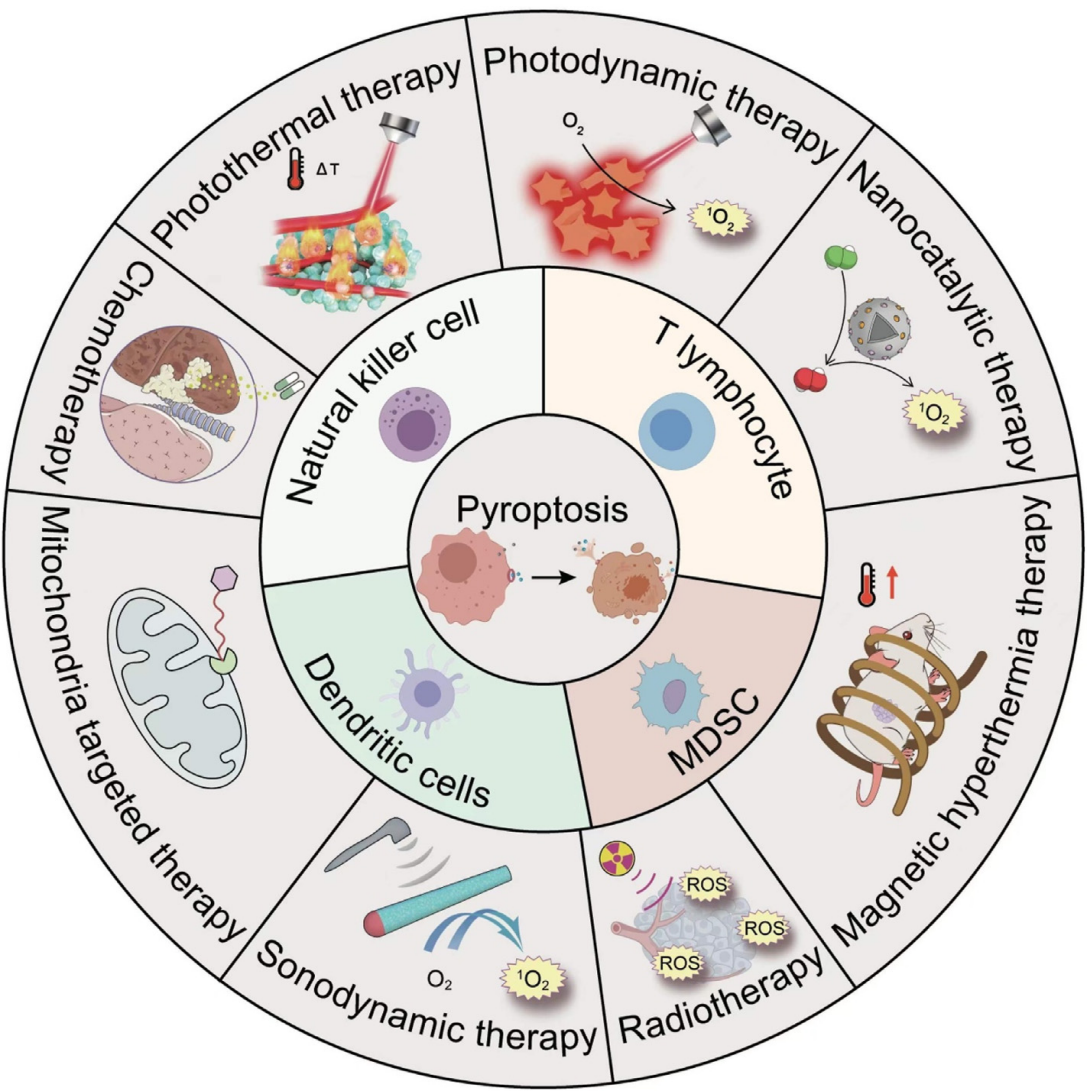
Schematic illustration of different modes of anti-tumor therapy strategies.

To attain early detection, diagnosis, and treatment of cancer, a combination of multiple tumor immunotherapies and nanomedicine is being employed to efficaciously impede tumor growth and migration, as well as enhance the rate of tumor remission^2^. This review provides an overview of the underlying mechanisms of pyroptosis and the signaling pathways that trigger this process, as well as the interplay between pyroptosis and immunity. Novel tumor immunotherapy possesses the benefit of precisely targeting cancerous cells. Furthermore, this review consolidates recent advancements in the utilization of novel nanomedicines for various tumor treatment modalities, including photodynamic therapy (PDT), photothermal therapy (PTT), chemotherapy, mitochondria-targeted therapy, nanocatalytic therapy, sonodynamic therapy (SDT), radiotherapy, magnetic hyperthermia therapy (MHT) and other approaches that activate pyroptosis *via* distinct mechanisms. Finally, the article paper is summarized to discuss the nanomedicine-induced pyroptosis in the future development of nanomedicine and to explore the biosafety and development of nanomedicine in tumor immunotherapy (Scheme 1).

## 2 The significance of pyroptosis for enhancing tumor immunotherapy

### 2.1 Discovery and mechanisms of pyroptosis

In 1986, Friedlander found that anthrax lethal toxin-induced cell death in mouse primary macrophages^3^. In 1992, Zychlisky et al.^4^ defined the characteristics of cell death found in macrophages infected with *Shigella flexneri* as apoptosis. In 2000, this group reported that infected macrophages were insensitive to conventional apoptosis but responded normally to other apoptotic stimuli. Next, more studies showed that the cysteinyl aspartate specific proteinase 1 (caspase-1)-dependent cell death mechanism was different from traditional apoptosis, and it is a new type of lytic cell death mode^5^. Pyroptosis is characterized by both apoptosis and pyroptosis and can lead to apoptosis-like DNA breaks and chromatin condensation. Previously, researchers used apoptosis to define the phenomenon of inflammatory cell death. The concept of pyroptosis was formally proposed by Brennan MA and Cookson BT in 2001, which was the first time it was described^6^^,^^7^.

Programmed cell death (PCD) is an actively ordered form of death initiated by gene regulation^8^. To maintain a stable internal environment, PCD can play a transitional role in the natural death of cells by removing abnormal cells from the tissue environment. PCD includes apoptosis, necroptosis, autophagy, pyroptosis, and ferroptosis, among others. Pyroptosis is a PCD mode that destroys cell membranes and releases the cellular contents to induce cell death mediated by gasdermin protein. Initially, pyroptosis was thought to be an inflammatory cell death caused by caspase-1 binding to the inflammasome and activating interleukin-1*β* (IL-1*β*)^9^, ^10^, ^11^. With the in-depth exploration of the pyroptosis signaling pathway, many studies have found that this form of cell death is not entirely determined by the caspases of apoptosis and inflammation but is determined instead by gasdermin protein^12^, ^13^, ^14^, ^15^. The gasdermin protein family has membrane perforation activity, including gasdermin A/B/C/D (GSDMA/B/C/D), gasdermin E (GSDME/DFNA5), and DFNB59 (PJVK). Among them, GSDMA/B/C/D and GSDME are composed of an N-terminal pore-forming domain (PFD) and a C-terminal repressor domain and use a caspase-mediated perforation mechanism to induce pyroptosis^16^^,^^17^. When an external pathogen invades the body to activate the signal, caspase proteins or granzymes cleave gasdermin protein to separate the N-terminus and C-terminus. Subsequently, the N-terminal PFD oligomerizes on the cell membrane and forms pores, releasing inflammatory molecules and causing pyroptosis of target cells^18^.

Different cell death modes have certain differences from the characteristics of pyroptosis but have important significance and connection in anti-tumor treatments. Compared with pyroptosis, apoptosis is an ordered death process regulated by apoptosis-related genes, which is characterized by DNA breakage, cell shrinkage, intact membrane structure and organelles, and formation of apoptotic bodies^19^. Apoptotic caspases are categorized as initiating caspases-8, caspase-9, and caspase-10 and executing caspases-3, caspase-6, and caspase-7. Necroptosis has similar necrotic morphology as pyroptosis^20^. Cells undergoing necroptosis show cell swelling, plasma membrane pore formation, and membrane rupture and release a variety of damage-associated molecular patterns and cytokines to trigger inflammatory responses^21^. Autophagy is a mode of cell death in which DNA degradation occurs and is distinguished from pyroptosis by the morphologic features that exhibit cytoplasmic vacuolization, the structural integrity of membranes, formation of autophagic vesicles, and removal of substances *via* lysosomes. Ferroptosis is caused by iron overload and reactive oxygen species (ROS) dependent accumulation of lipid peroxides, which leads to smaller cellular mitochondria, increased membrane density, reduced or absent cristae, and rupture of the outer membrane^22^. Relevant studies have shown that CD8^+^ T cells can induce and promote both pyroptosis and ferroptosis, which will be of great significance in the study of activation and differentiation of anti-tumor immune cell function^23^. In addition, it is worth mentioning that PANoptosis is an inflammatory PCD regulated by the PANoptosome complex and has the important features of pyroptosis, apoptosis, and necroptosis. Cell death types are difficult to completely differentiate by simply observing the cell morphology, and it is necessary to distinguish it from pyroptosis by multiple indicators and mechanisms^24^.

### 2.2 Signaling pathways of pyroptosis

#### 2.2.1 Canonical pathway

A functional inflammasome is initiated by germline-encoded pattern recognition receptors (PRRs) that detect pathogen-associated molecular patterns (PAMPs), danger-associated molecular patterns (DAMPs), and homeostasis-altering molecular processes. A PRR recognizes DAMPs or pathogen-associated molecular patterns when external pathogenic factors are present. Subsequently, PAMPs and DAMPs are recognized by sensor proteins, including nod-like receptor protein 1 (NLRP1), NLRP3, NLRC4, absent in melanoma 2 (AIM2), and pyrin^25^^,^^26^. Among them, the adaptor protein ASC binds to pro-caspase-1 and activates caspase-1. Activated caspase-1 can activate the precursors of IL-1*β* and interleukin-18 (IL-18) and release them to the cell membrane to produce an inflammatory response. GSDMD can be cleaved into two fragments: the C-terminal domain and the N-terminal domain. Subsequently, the N-terminal domain binds to phospholipoprotein on the cell membrane, forming pores and releasing cellular contents to induce pyroptosis^27^^,^^28^.

#### 2.2.2 Noncanonical pathway

In the non-classical pathway, lipopolysaccharide (LPS) directly mediates the pyroptosis process triggered by caspases-4/5 in humans and caspase-11 in mice. Activated caspase-4/5/11 cleaves GSDMD into a C-terminal domain and an N-terminal domain. The N-terminal domain binds membrane phospholipids and causes the cell membrane to form pores to release the cellular contents. At the same time, GSDMD-N leads to a K^+^ efflux to activate the NLRP3 inflammasome and caspase-1^29^^,^^30^. However, caspase-4/5/11 is not involved in the activation of pro-IL-1*β* and pro-IL-18. The maturation and release of cytokines are indirectly regulated by the NLRP3/caspase-1 pathway. It is worth noting that NLRP3, a member of the NLR protein family, is the main inflammatory complex in pyroptosis. Because of the way the NLRP3 inflammasome is involved, activated caspase-11 can indeed induce a small amount of IL-1*β* secretion^31^^,^^32^.

#### 2.2.3 Other pathways

**Scheme 2:**
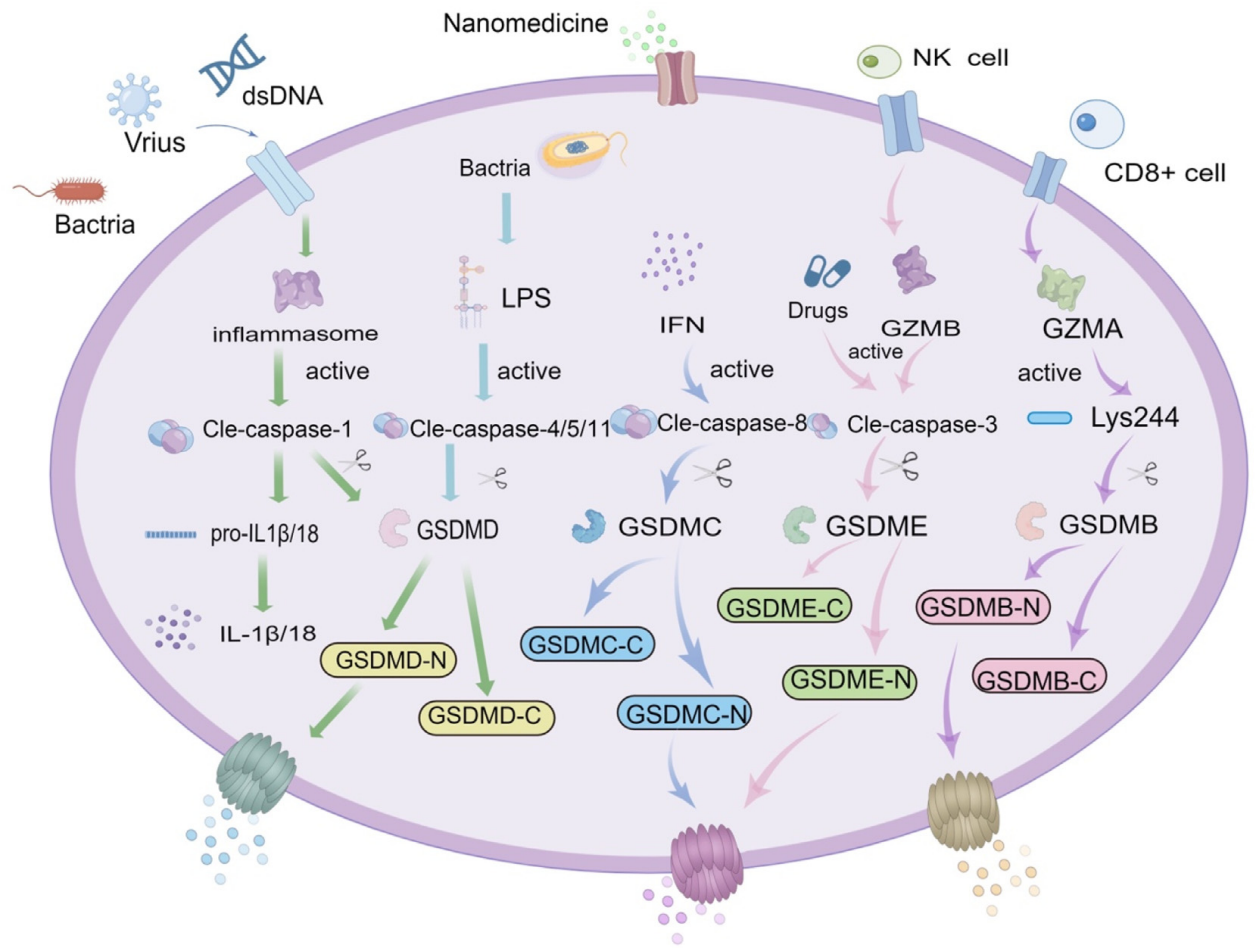
Schematic illustration of the pyroptosis signaling pathways.

Besides the two above-mentioned main pathways, there are other alternative means to induce pyroptosis, including 1) other cysteine aspartic proteases can activate caspase-8 or caspase-3 to cleave GSDMC to form transmembrane channels under the action of interferons and ultimately mediate pyroptosis^33^, ^34^, ^35^; in addition, caspase-3 can be triggered to cleave GSDME protein to induce pyroptosis in the case of chemotherapy. 2) Granzymes are expressed in cytotoxic T lymphocytes (CTLs) and natural killer cells (NK cells), which cleave specific substrates in their target cells^36^. Activated caspase-8 or granzyme B (GzmB) cleaves GSDME, while caspases-1 and granzyme A cleave GSDMB. Granzyme A cleaves GSDMB at the Lys site of the interdomain ligase (Scheme 2)^37^^,^^38^.

### 2.3 Relationship between pyroptosis and immunity

Immunogenic cell death (ICD) is a process in which the body produces an anti-tumor immune response driven by stress, which can be transformed from being non-immunogenic to immunogenic^39^^,^^40^. Pyroptosis is an ICD process. When tumor cells are stimulated by external stimuli, the molecular mechanism of pyroptosis is induced, and DAMPs are released. The extracellular calreticulin releases signals to immune cells, and ICD occurs. Subsequently, high mobility group protein B1 (HMGB1) is released extracellularly and binds to PRRs expressed by dendritic cells (DCs) to activate the corresponding signaling pathway^41^. In addition, Adenosine triphosphate (ATP) released into the extracellular space regulates immune cell infiltration, causing a series of immune responses. Pyroptosis promotes the infiltration of immune cells into the immunosuppressive TME by producing pro-inflammatory cytokines, such as IL-1*β* and IL-18, which can be used for anti-tumor therapy^42^^,^^43^.

GSDMD protein in the gasdermin family induces pyroptosis through classical and noncanonical pathways. By activating caspase-1/4/5/11 to cleave GSDMD protein to promote cell swelling, proinflammatory immune factors are released to enhance the immune system. As the first protein to be found to interact with pyroptosis, the role of the GSDMD protein is clearer than that of others in the immune mechanism of pyroptosis. In addition, the cleavage of caspase-3-related sites by GzmB can activate the GSDME protein as a tumor suppressor. Related studies have shown that the phagocytosis of tumor-associated macrophages was enhanced by inducing pyroptosis of GSDME in tumors^44^. At the same time, tumor-infiltrating NK and CD8^+^ T killer lymphocytes are increased to inhibit tumor growth. Activation of GSDME in the TME transforms “cold” tumors that cannot be recognized by the immune system into controllable “hot” tumors^45^. Apoptosis is converted to pyroptosis, which inhibits tumor cell proliferation and metastasis^46^. Gasdermin protein plays an important role in anti-tumor immune treatment and provides ideas for further exploring the therapeutic strategy of pyroptosis in immune mechanisms.

Pyroptosis induced by gasdermin protein is closely related to tumor development, but its anti-tumor immune mechanism remains to be further studied. Current studies have found that activation of pyroptosis changes the TME to promote cancer immunotherapy and affects tumor growth and metastasis. For example, the rapid growth of tumors leads to vascular abnormalities, which leads to hypoxia in the tumor. This then causes an imbalance of the antioxidant system in the TME. Pyroptosis induces oxidative stress in cells to increase the immunogenicity of tumors. This will eventually lead to immunogenic death of tumor cells^47^. ROS acts as an agonist of NLRP3 and reprograms TME. Its inflammasome activates caspase to induce pyroptosis to improve the immunosuppressive TME and induce ICD to achieve anti-tumor immunity. Tumor cells have a strong antioxidant function and can scavenge ROS to adapt to the oxidative environment to avoid cell death. Nanomedicines can stimulate the antioxidant system and increase the ROS level to inhibit tumor growth. The use of new nanomedicines can produce a large amount of ROS to regulate the redox equilibrium. In the process of tumor treatment, increasing ROS levels by this method can induce oxidative stress in cells and promote the release of cell damage-related molecular pattern^48^^,^^49^.

## 3 Nanomedicine-induced pyroptosis of tumor therapy

Nanomedicine, a specialized area of medicine that utilizes nanotechnology and nanomaterials for disease prevention, diagnosis, and treatment, has seen significant research in recent years focusing on the induction of pyroptosis in tumor therapy^50^. The sizes and shapes of nanomedicines play crucial roles in their accumulation and diffusion within tumors, as well as in the activation of pyroptosis^51^^,^^52^. Some cases of how different sizes and shapes of nanomedicines influence the pathways of pyroptosis have been reported in literature^53^^,^^54^. For example, the sizes of graphene oxide (GO), a representative two-dimensional (2D) nanomaterial, play a key role in inducing pyroptosis in hepatocytes, with large-sized GO (500–2000 nm) inducing pyroptosis more strongly than small-sized GO (50–200 nm)^55^. In addition, silica nanoparticles (NPs) can penetrate the blood–brain barrier and enter the central nervous system. In neurotoxicity studies, 50–300 nm silica NPs induced pyroptosis *via* the GSDMD signal pathway. Smaller silica NP-induced pyroptosis had the highest cytotoxicity and produced higher IL-1*β* and cell swelling^56^. Therefore, the sizes and shapes are vital factors in the design of nanomedicine for pyroptosis-mediated tumor therapy, and developing nanomedicine with different structural features could enrich existing tumor treatment strategies to enhance the efficacy of tumor immunotherapy^57^^,^^58^.

### 3.1 Photodynamic therapy (PDT)

**Figure 1:**
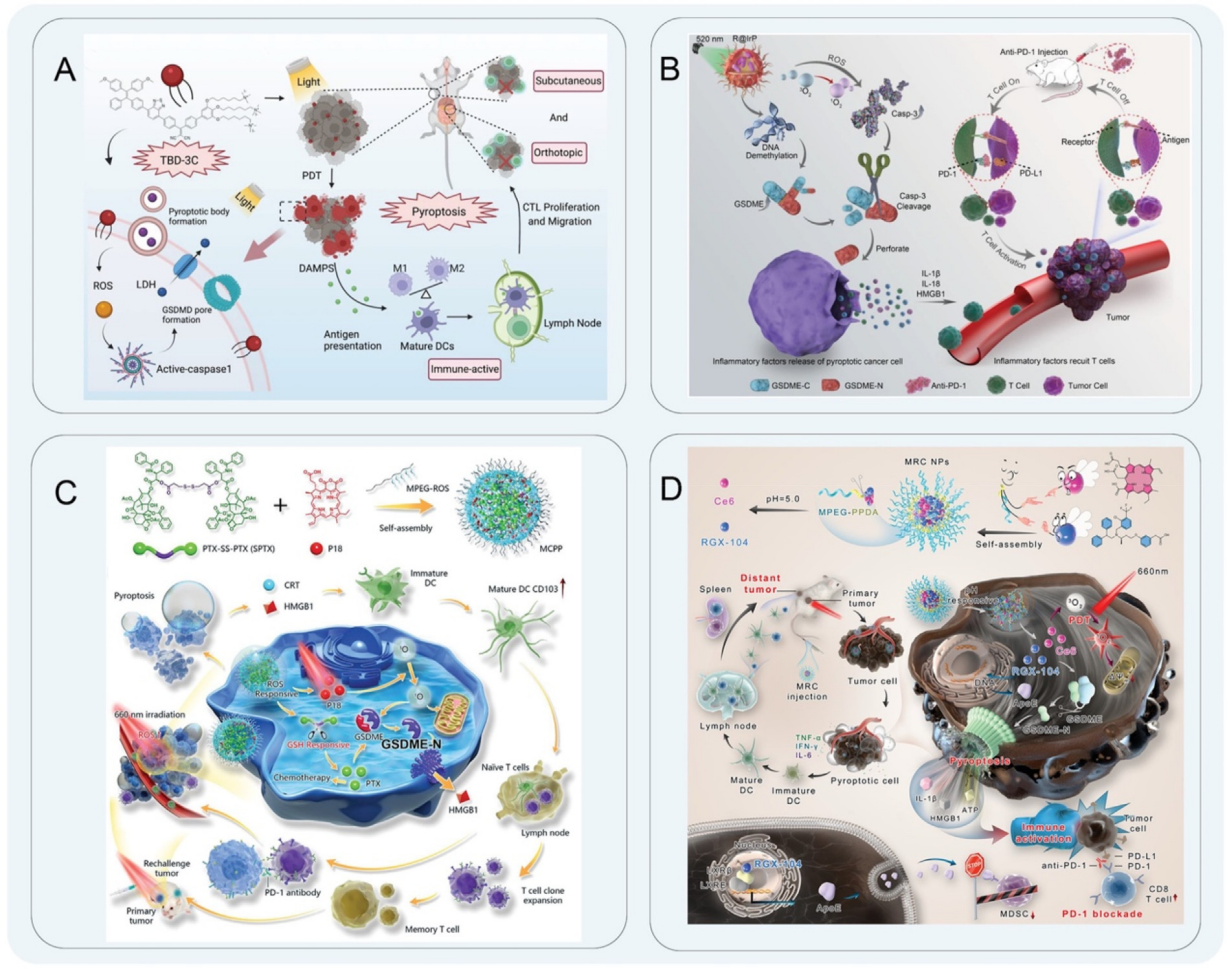
Schematic diagram of PDT. (A) Schematic illustration of TBD-3C activates inflammatory caspases-1 cleavage GSDMD to improve the efficacy of immunotherapy for pancreatic cancer. Reprinted with the permission from Ref. 64. Copyright © 2018 Copyright 2020 Wiley. (B) Nano-agonists stimulate caspase-3 to up-regulate the expression of GSDME protein. Reprinted with the permission from Ref. 68. Copyright © 2023 Wiley. (C) The synthesis of MCPP and GSDME protein performs pyroptosis to promote anti-tumor immunity. Reprinted with the permission from Ref. 72. Copyright © 2021 Wiley. (D) Hydrophobic MRC NPs activate caspase-3 to induce pyroptosis for anti-tumor immunity to alleviate immunosuppression. Reprinted with the permission from Ref. 81. Copyright © 2022 Wiley.

PDT is an optical therapeutic modality that involves the utilization of a photosensitizer to absorb the appropriate wavelengths under laser light, resulting in a photochemical reaction that leads to the destruction of malignant cells. PDT is a direct cancer cell-killing strategy with few side effects in normal tissues due to its non-invasive and spatiotemporally controllable nature^59^^,^^60^. Photosensitizers, as intermediates in PDT, are activated by light to produce biotoxic singlet oxygen to kill tumor cells^61^^,^^62^. The stimulation of ROS generated during PDT treatment can initiate anti-tumor immunity by inducing ICD to form effective lysosomal damage and inflammasomes to induce pyroptosis of tumor cells^63^. Wang et al.^64^ designed a novel membrane-targeted photosensitizer (TBD-3C) for the treatment of pancreatic cancer. In their study, PDT was used to reverse the inhibition of the TME to induce pyroptosis and activate anti-tumor immunity. This strategy activated inflammatory caspase-1 to cleave GSDMD and release the N-terminal domain. This caused cell swelling and membrane rupture, which released the cell contents to stimulate the immune response, effectively promoting cascade amplification. Pyroptosis induced by TBD-3C stimulated macrophages to M1 polarization, induced maturation of DCs, and activated CD8^+^ cytotoxic T-lymphocytes, which significantly enhanced anti-tumor immunity (Fig. 1A). Lu et al.^65^ reported an organic photo-immune activator NBS-1MT, the combination of photosensitizer and indoleamine 2,3-dioxygenase (IDO) inhibitor^66^. NBS-1MT triggered pyroptosis by activating the caspase-1/GSDMD signaling pathway, leading to the suppression of primary and distant tumor growth. The induction of pyroptosis by NBS-1MT notably enhanced the infiltration of CTLs within both primary and distant tumor sites. Moreover, the secretion levels of tumor necrosis factor-alpha (TNF-*α*), interferon-gamma (IFN-*γ*), interleukin-6 (IL-6), and interleukin-12 in serum were significantly elevated as a result of NBS-1MT treatment.

The tumor suppressor GSDME converts non-inflammatory apoptosis into inflammation-mediated pyroptosis through caspase cleavage^67^. The expression of GSDME enhanced the phagocytosis of tumor-associated macrophages and the number and function of tumor-infiltrating NK cells and CD8^+^ T lymphocytes. Zheng et al.^68^ synthesized a photoactivated iridium-based photosensitizer (IrP) and self-assembled with a GSDME protein methyltransferase inhibitor (RG108). The resulting nanoagonist increased GSDME expression by RG108 treatment, leading to caspase-3/GSDME-mediated pyroptosis^69^. Additionally, light-induced pyroptosis in combination with anti-programmed death 1 (*α*PD-1) therapy demonstrated efficacy in anti-tumor photoimmunotherapy. The release of pro-inflammatory factors from pyroptotic cells altered the inflammatory microenvironment, attracting immune cells to eliminate tumor cells. This process activated CD8^+^ CTLs, enhanced PD-1 expression, and promoted the maturation of DCs. Results of the flow cytometry experiments demonstrated a notable augmentation in the population of CD3^+^, CD4^+^ and CD8^+^ T cells, which successfully activates the immune system and recruits immune cells to attack the tumor (Fig. 1B). Wang et al.^70^ synthesized the prodrug TPRA–SS–DAC (TSD) by covalently coupling decitabine (DAC) and photosensitizer TPRA, which was released in the highly reduced tumor cell environment and increased the expression of GSDME and programmed death-ligand 1(PD-L1). The light was used to activate pyroptosis to promote the maturation of DCs and increase tumor infiltration of CTL. Based on the synthesized prodrug, a ligand-switchable nanocarrier (LSN-D) modified with PD-L1 blocking peptide (DC-DPPA) was used to develop TME dual-responsive nanomedicine (TSD@LSN-D). It combines epigenetic modulators with PDT to induce pyroptosis and promote anti-tumor immunity^71^. Moreover, the pH-responsive nanoshell provided *in situ* exposure of the DC-DPPA peptide to tumors and achieved maximum blockade of PD-L1 receptors on tumor cells. TSD@LSN-D triggered immunogenic pyroptosis induced higher levels of CD8^+^ T cell infiltration, high GzmB expression, and significantly elevated serum IL-6, IFN-*γ*, and TNF-*α* levels. Xiao et al.^72^ loaded a ROS/glutathione (GSH) dual-responsive multifunctional chemical photodynamic nanomedicine (MCPP) with a high paclitaxel concentration and the photosensitizer purine 18. Under combined chemotherapy-PDT, the successful release of the nanomedicine was achieved, and the MCPP induced DSDME-dependent pyroptosis after laser irradiation^73^. GSDME is cleaved by caspase-3 and releases the GSDME-N domain. Subsequently, the GSDME-N domain forms membrane pores, leading to cell swelling and rupture and the release of tumor antigens and DAMP. Up-regulation of CD103 expression induces maturation of DCs, an increase in the proportion of CD3^+^ T cells in lymph nodes, and promotes T cell clonal expansion, which triggers an adaptive immune response (Fig. 1C).

Chlorin e6 (Ce6), a photosensitizer, is frequently utilized in the development of novel nanomedicines for PDT owing to its remarkable ability to produce singlet oxygen with great efficacy^74^^,^^75^. The Ce6 has a strong absorption spectrum at 600–700 nm and 400–500 nm, and the absorption in the Q-band is higher than that of other porphyrins. Therefore, it has the advantages of selective high tumor uptake, strong photosensitive oxidation characteristics, and low toxicity and is one of the nanomedicines that deserves attention at this stage^76^^,^^77^. Li et al.^78^ constructed an intelligent nano-tuning platform by connecting Ce6 and the quencher QSY21 to a prepared amphiphilic copolymer of ultra-pH sensitive materials. The nanoplatform was composed of nine nanomedicines ANPS libraries with pH transition values ranging from 5.3 to 6.9 and was combined with a PDT strategy. Through the endosomal maturation pathway, nanomedicines were precisely controlled to enter the endocytic region in a non-invasive manner to target and activate pyroptosis. It was found that early endosomes produced oxidative stress through lipid peroxidation-specific activation of phospholipase C signal transduction mediated by ANPS. Subsequently, caspase-3-mediated GSDME cleavage and pyroptosis were induced. Myeloid-derived suppressor cells (MDSCs), as negative regulators of immunity in the TME, inhibit T cell activation and a variety of immune cell activities^79^^,^^80^. The application of new pyroptosis inducers reduced the expression level of MDSCs and improved the immune effects of the TME in promoting pyroptosis. To this point, Qiu et al.^81^ prepared a hydrophobic MRC nanoparticle as a pyroptosis inducer for anti-tumor immunotherapy by using the pH-responsive carrier MPEG-PPDA loaded with the immune agonist RGX-104 and photosensitizer Ce6. Immunostimulant RGX-104 acted on liver X nuclear hormone receptors (LXR*α* and LXR*β*) to stimulate apolipoprotein E (ApoE) expression, which reduced the level of MDSCs and improved the TME to reverse immunosuppressive activity (Fig. 1D). In an established mouse tumor model, cleaved caspase-3, GSDME-N, and ApoE expression was upregulated. In tumor cells, PDT mediated by Ce6 stimulated ROS production and induced pyroptosis *via* GSDME. During this process, there was an increase in the secretion of TNF-*α*, IFN-*γ*, IL-6, and IL-1*β*, as well as an upregulation in the expression of CD80^+^ and CD86^+^ cells. Additionally, CTLs secreted perforin and GzmB, which led to an enhancement in the anti-tumor immune response. Zhou et al.^82^ also designed a tumor-specific prodrug (CANP) that combined Ce6 with the heat shock protein 90 inhibitor 17-allylamino-demethoxy-geldanamycin (17-AAG) to induce pyroptosis. Under 660-nm laser irradiation, this ROS-mediated signaling pathway activator activated caspase-9 and caspase-3 cleaved GSDME and ruptured the cell membrane to produce pyroptosis. Among them, 17-AAG not only induced ROS accumulation to enhance the efficacy of PDT but also significantly enhanced tumor targeting and attenuated 17-AAG-induced hepatotoxicity. At the same time, tumor-infiltrating lymphocytes were increased, and MDSCs in tumors were decreased. Immunomodulation combined with PDT can increase immunogenicity to promote anti-tumor immunity and inhibit primary and distant tumor growth. Hu et al.^83^ prepared a nanovesicle with Golgi-targeting ability (ChS-Ce6) to activate NLRP3, and the Golgi-targeting ability of chondroitin sulfate was combined with the photosensitizer Ce6 to induce pyroptosis for anti-tumor immunity in PDT. Further evaluation of the immune effects of ChS-Ce6-induced pyroptosis showed a significant increase in the proportion of CD80^+^CD86^+^ cells in tumor-draining lymph nodes and a markedly higher infiltration of CD4^+^ and CD8^+^ T cells in the tumor tissues. Assessment of distant metastasis and recurrence of the tumor showed an increase in effector memory T cells.

Photodynamic strategies can induce pyroptosis using not only photosensitizers but also photoactivated complexes as pyroptosis inducers in combination with PDT. Ling et al.^84^ designed a photoactive complex (Pt1 and Pt2) as a pyroptosis inducer to achieve controllable treatment of malignant tumors. In the process of PDT, platinum (II) triphenylamine complexes acted on two pathways to cause an anti-tumor immune response. The complexes modulated the immune response by triggering ICD through the action of metal ions. Upon exposure to light at 425 nm, the complex not only caused damage to mitochondrial and nuclear DNA but also initiated GSDMD-mediated pyroptosis by activating the cGAS-STING pathway. This led to the release of dsDNA into the cytoplasm, resulting in the maturation of DCs and a two-fold increase in the proportion of CD8^+^ T cells and CD4^+^ T cells.

### 3.2 Nanocatalytic therapy

**Figure 2:**
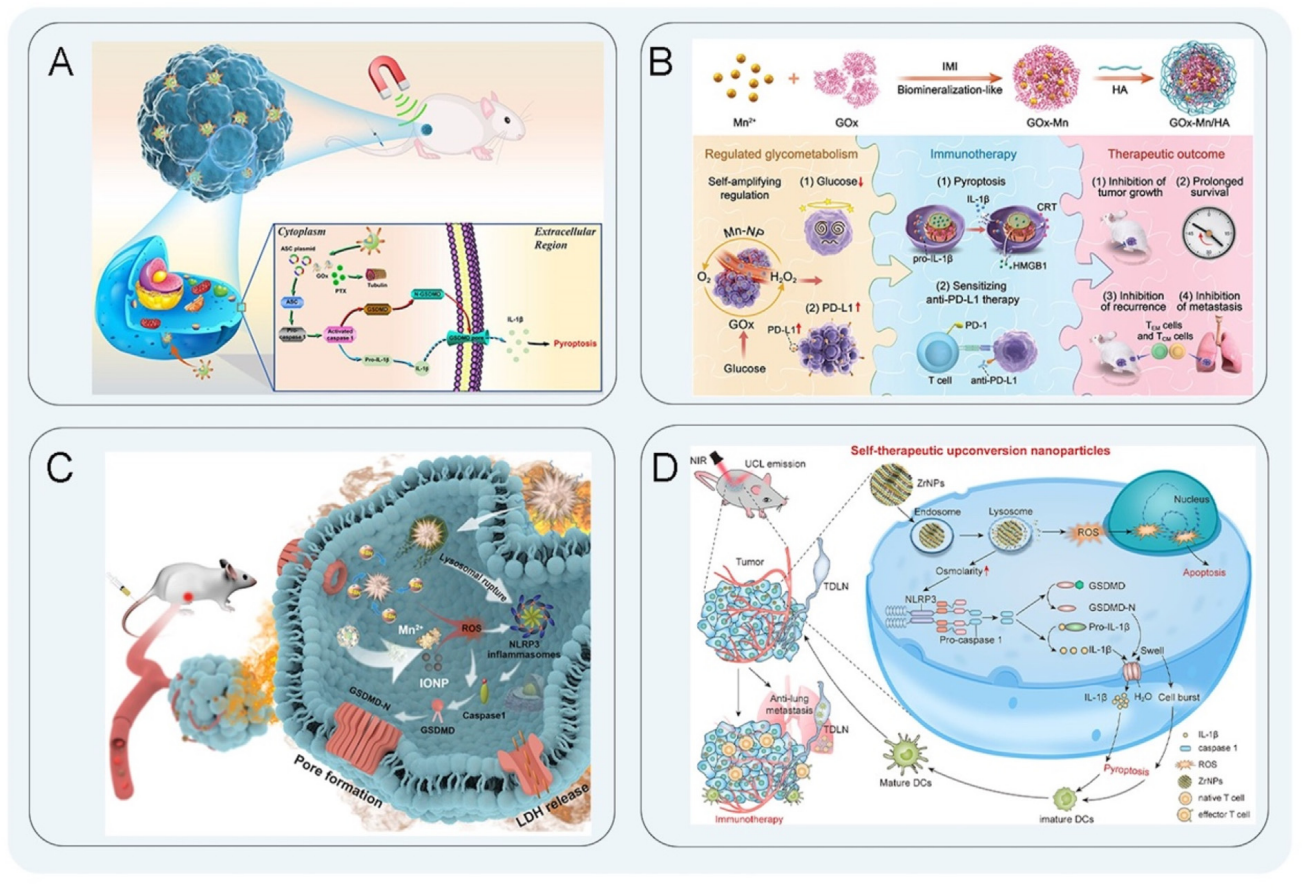
Nanocatalytic therapy schematic diagram. (A) Schematic illustration of the smart drug delivery system mediates pyroptosis-starvation-chemotherapy combination therapy for breast cancer by ASC activating caspase-1. Reprinted with the permission from Ref. 89. Copyright © 2022 American Chemical Society. (B) GSDMD cleavage mediated pyroptosis mediated by dual enzyme nanomedicines through glucose metabolism regulation. Reprinted with the permission from Ref. 91. Copyright © 2022 Wiley. (C) The pyrolytic agent pierced lysosomes to release Mn^2+^ and IONPs and synergistically activated NLRP3 and caspase-1 to induce pyroptosis. Reprinted with the permission from Ref. 100. Copyright © 2021 Wiley. (D) ZrNPs regulate homeostasis and orderly activation of caspase-1 to induce pyroptosis and promote maturation of DCs. Reprinted with the permission from Ref. 103. Copyright © 2021 American Chemical Society.

Nanocatalytic therapy is a therapeutic approach that involves the activation of a chemical catalytic reaction of nanomedicines within the body through the application of external physical or chemical stimuli, resulting in the induction of oxidative stress within tumors^85^. In tumor therapy, nanomedicines activate the molecular pathway of pyroptosis and increase the level of ROS. Nanocatalytic reactions can effectively cleave GSDMD protein and lead to pyroptosis^86^^,^^87^. Tumor cells have a strong ability for glucose uptake in the TME, which leads to limited glucose supply to anti-tumor cells. The consumption of glucose oxidase promotes T cells, DCs, and other immune factors to better stimulate the immune response^88^. Based on this, several studies have found that reducing the supply of glucose by regulating glucose metabolism through nanosystems induces greater pyroptosis and stimulates anti-tumor immunity. Liu et al.^89^ prepared nanomedicine (PTX-ASC-GO@MP NPs) by using poly (lactic*-co-*glycolic acid) (PLGA) to encapsulate iron oxide nanomedicines (IONPs) and apoptosis-associated speck-like protein containing a caspase recruitment domain (ASC) plasmid and paclitaxel. The combination of pyroptosis-starvation chemotherapy effectively treated breast cancer. Glucose oxidase is a starvation agent that can consume glucose in the tumor to reduce the energy supply for the tumor^90^. In addition, ASC initiated pyroptosis *via* the caspase-1/GSDMD signaling pathway and released high levels of IL-1*β* and TNF-*α*, which activates T cells with the increase of activated T helper cells and CTLs (Fig. 2A). Zhang et al.^91^ used a two-enzyme synthesis strategy and hybridized manganese (Mn)-containing nanozymes and glucose oxidase to construct dual-enzyme active nanomedicines with tumor cell glucose metabolism. The nanomedicines were exposed from the endoplasmic reticulum to the cell surface *via* endoplasmic reticulum calcium-binding proteins. GSDMD promoted cell membrane rupture, leading to pyroptosis, and released HMGB1 from the nucleus to the outside of the cell, causing the tumor cells to express more PD-L1^92^. In addition, effector memory T (T_EM_) cells and central memory T (T_CM_) cells exhibit superior anti-tumor immune responses. The proportions of CD4^+^ T_EM_ cells, CD8^+^ T_EM_ cells, CD4^+^ T_CM_ cells, and CD8^+^ T_CM_ cells were all significantly elevated, while glucose consumption was amplified in the cell cycle, leading to the dual effects of glucose metabolism and tumor immunotherapy (Fig. 2B). Li et al.^93^ constructed a ROS-responsive nanoreactor based on polyion complex vesicles, which initiated immunogenic self-pressurized catalytic glucose oxidation through induced pyroptosis. The nanoreactor also induced pyroptosis through oxidative stress induction and glucose starvation. Its vesicle structure protects the glucose oxidase catalyst in the TME to maintain long-term activity to kill tumor cells.

Regulating glucose metabolism and combining it with immunotherapy offers promising anti-tumor immunotherapy, but the metabolic pathways of tumor cells during pyrolysis need to be explored in greater depth. In another study, glucose oxidase was used to propose a new concept for pyroptosis adjuvants. Ding et al.^94^ designed a sea urchin-like manganese oxide loaded with glucose oxidase. In the TME, a large amount of ROS was produced by a cascade of catalytic reactions, and then, caspase-1 was activated to cleave GSDMD, which released lactate dehydrogenase (LDH) enzyme to induce pyroptosis to achieve pyroptosis-induced immune activation. Pyroptosis adjuvants (PTAVs) produced a strong immune response during tumor treatment with increased proportions of CD3^+^ T cells, CD8^+^ T cells, CD8^+^/CD4^+^ and DCs, effectively activating cellular immunity. When a PTAV binds to an antigen or is injected into the body before an antigen, it is used as a repository for effective antigen delivery and to enhance antigen uptake, thus improving the specific immune response of immunotherapy. There are still many challenges for therapeutic tumor vaccines^95^^,^^96^. However, the rapid development of nanomedicines has made it possible to precisely tailor nano PTAVs for modern vaccines with a predetermined immune response.

At present, lysosomes have become an important target for anti-tumor therapy^97^. Many nanomedicines can activate GSDMD protein pyroptosis by targeting lysosomes through endocytosi^98^, but the immune regulation effect of nanocatalytic therapy in the field of pyroptosis needs to be further studied. Ploetz et al.^99^ used liposome-encapsulated MIL-100 (Fe) MOF NPs to enter cells by endocytosis. The acidified nanomedicine composed of iron and trimesic acid could be degraded and ruptured to release a large amount of iron in the acidic TME. Subsequently, caspase-1 activated the release of GSDMD protein and IL-1*β*, leading to cell swelling and membrane rupture, inducing pyroptosis. By stimulating lysosomal rupture and releasing ions to induce Fenton or Fenton-like reactions, ROS was generated in the tumor tissues and led to pyroptosis. Nadeem et al.^100^ designed a pyrolysis agent to activate tumors, which was composed of IONPs in the core and manganese dioxide spines on the outside. The pyrolysis agent efficiently accumulated in the tumor area. The special spiny structure of the nanomedicines pierced the lysosomes and released Mn^2+^ and IONPs, which cooperated with increased ROS levels for NLRP3 inflammasome activation and caspase-1 activation. Subsequently, the GSDMD protein was cleaved and perforated the cell membrane to rupture, inducing pyroptosis (Fig. 2C). Wang et al.^101^ constructed a gasdermin nanoplatform (M-CNP/Mn@pPHS) by incorporating a pyroptosis-inducing plasmid (pPHS) into Mn-containing calcium carbonate NPs, which could evade lysosomal degradation of the plasmid. M-CNP/Mn@pPHS could generate a strong anti-tumor immune response by inducing pyroptosis, which resulted in high proportions of mature DCs and CD8^+^ T cells and the release of a large number of IFN-*γ*, leading to the activation of systemic immune response and ultimately inhibition of tumor growth^102^.

In other nanocatalytic therapies, upconversion NPs had the effect of being specifically activated in the TME with the advantage of reducing cost and increasing efficiency as a pyroptosis inducer. Ding et al.^103^ constructed a type of K_3_ZrF_7_:Yb/Er upconversion NPs (ZrNPs) as pyroptosis inducers for cancer immunotherapy. ZrNPs dissolved in cancer cells and released large amounts of K^+^ and [ZrF_7_]^3‒^ to induce increased ROS, resulting in an intracellular osmotic pressure surge and homeostasis imbalance. Furthermore, the ROS induced pyroptosis *via* the caspase-1/GSDMD signaling pathway, resulting in cell swelling and cytolysis. The proportions of CD3^+^ T cells, CD8^+^ T cells, CD8^+^/CD4^+^, and DCs of the ZrNPs-treated group were multiplicative relative to the control group. The findings demonstrate that ZrNPs-induced pyroptosis could inhibit tumor growth and pulmonary metastasis, indicating robust anti-tumor immunity (Fig. 2D). In addition, studies have found that the mitochondrial respiratory inhibitor oligomycin A (OA) effectively inhibited mitochondrial function and induced *in situ* intracellular oxidative stress, ultimately inducing anti-melanoma apoptosis-pyroptosis conversion. Wang et al.^104^ designed a MOF (MIL101-NH_2_-Fe) by encapsulating OA into cyclic arginine-glycine-aspartic acid (c(RGDyC))-modified iron. The signaling pathway that overcame certain anti-apoptotic activity and induced an immune response against melanoma implied the occurrence of apoptosis and pyroptosis. Mitochondrial oxidative stress-activated caspase-3 to trigger apoptosis by cleaving poly (ADP-ribose) polymerase. At the same time, the activated caspase-3 could cleave the GSDME^DMLD^ site and induce pyroptosis with the formation of pores on the cell membrane. Subsequently, the cell contents flowed out of the cell, and bioactive cytokines were secreted, as well as the formation of macromolecular inflammasome complexes, and finally, pyroptosis was induced. The proportions of both CD8^+^ T cells and CD4^+^ T cells in the spleen of mice were significantly increased. Meanwhile, the population of regulatory T cells (Treg) inhibiting the immune response notedly decreased after treatment, indicating significant anti-tumor immunity effects.

### 3.3 Sonodynamic therapy (SDT)

**Figure 3:**
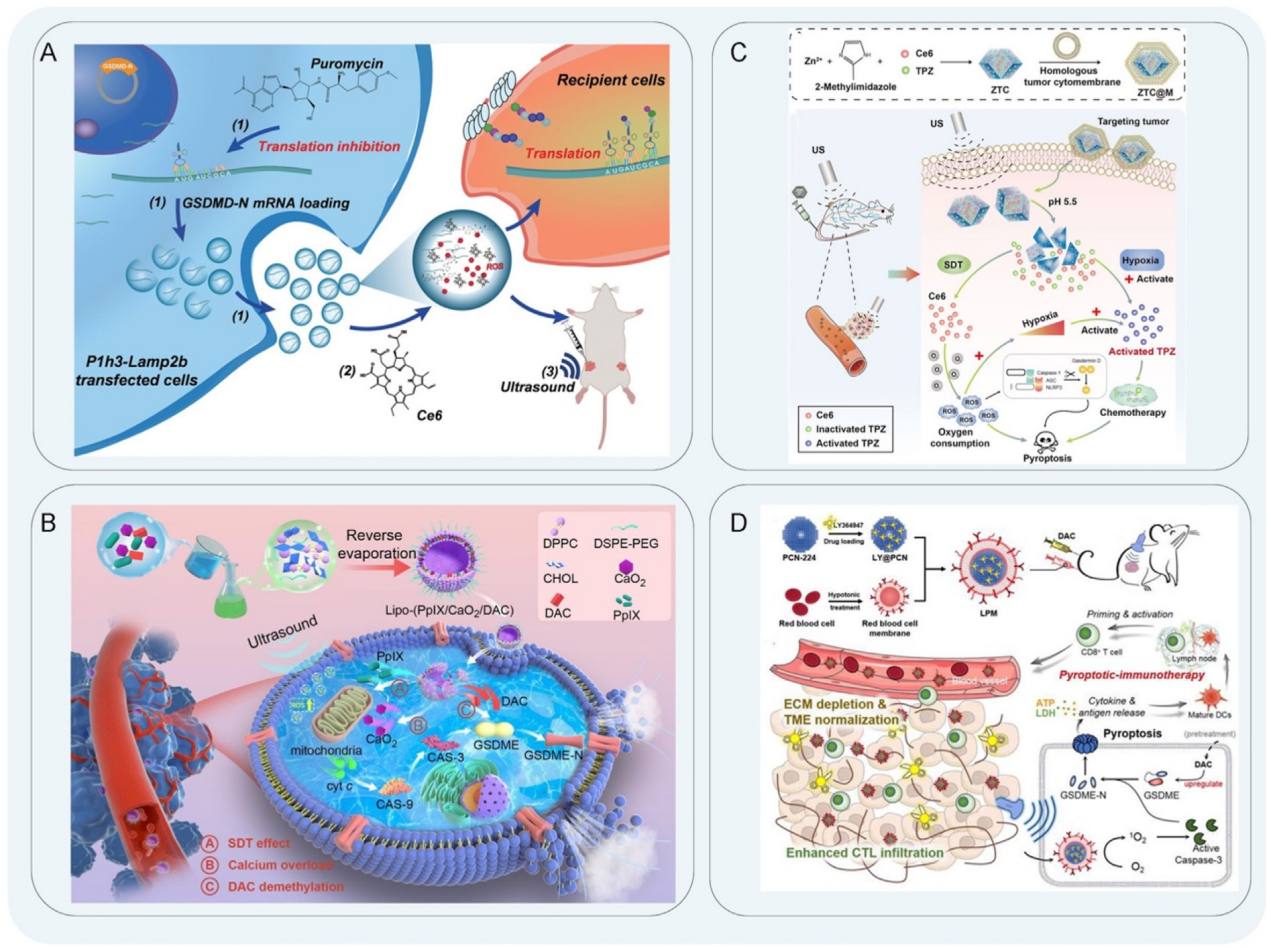
Schematic diagram of SDT. (A) Schematic illustration of EV^Tx^ was delivered into HER2^+^ cells and induced pyroptosis to inhibit tumor growth after SDT. Reprinted with the permission from Ref. 107. Copyright © 2023 Wiley. (B) SDT activates lipid (PpIX/CaO_2_/DA) to destroy mitochondrial Ca^2+^ buffering capacity, and GSDME is cleaved into the GSDME-N domain to induce pyroptosis. Reprinted with the permission from Ref. 108. Copyright © 2023 Elsevier. (C) ZTC@M NPs were synthesized and triggered by ultrasonic to activate caspase-1 to induce pyroptosis. Reprinted with the permission from Ref. 110. Copyright © 2022 Frontiers Media S.A (D) SDT produces a large amount of ROS to activate caspase-3 to induce pyroptosis and increase T cell infiltration. Reprinted with the permission from Ref. 109. Copyright © 2023 Ivyspring International.

SDT is a non-invasive modality for cancer treatment that induces cell death through the process of lysing deep-seated cancer cells *via* non-thermal ultrasound-activated sonosensitizers. Its main feature is its non-invasive, deep-tissue penetration ability. It has two dimensions, both time and space, for selective tumor cell killing^105^. SDT is a new non-invasive treatment method based on traditional treatment methods and PDT. The sonosensitizer synthesized using nanomedicines plays a key role^106^. The sonosensitizers used for the synthesis of nanomedicines provide sonodynamic anticancer action, such as sonoluminescence-mediated anticancer effects as ultrasound-responsive agents. Xing et al.^107^ encapsulated GSDMD-N mRNA inside an extracellular vesicle membrane with Ce6 embedded in the vesicle membrane and placed HER2 antibody on the vesicle surface as a new drug delivery carrier. After the nanomedicine delivery system (EV^Tx^) was delivered to HER2^+^ breast cancer cells, it was inactivated by SDT and diluted with puromycin to release GSDMD-N mRNA, which was translated in donor cells and induced pyroptosis (Fig. 3A). The high expression of GSDMD protein initiated pyroptosis and activated the immune system, which produced a strong tumor immune response and inhibited tumor growth, providing a nanoplatform for cancer immunotherapy. Zhang et al.^108^ designed a multifunctional nano-sonosensitizer protoporphyrin IX (PpIX) loaded with the calcium supplement CaO_2_ and DAC (lipo-(PpIX/CaO_2_/DAC)) to introduce Ca^2+^ into the cytoplasm to effect mitochondrial dysfunction and induce GSDME-dependent pyroptosis tumor therapy through calcium overload (Fig. 3B). DAC is a DNA methyltransferase inhibitor, which is often used to upregulate the expression of GSDME and lyse caspase-3 to form a cytotoxic GSDME N-terminal, which triggers cancer cell pyroptosis^109^. In addition, an elevated intracellular Ca^2+^ concentration promoted the release of mitochondrial cytochrome *c*, cleaved GSDME, and induced strong pyroptosis.

The sonosensitizer targeted at the tumor site is activated through precise ultrasound localization. Upon entering cells, sonosensitizers generate ROS, which can induce oxidative stress and ultimately lead to the destruction of tumor cells. Yu et al.^110^ synthesized a zeolitic imidazolate framework-8 (ZIF-8) for sonodynamic synergistic chemotherapy to induce pyroptosis by encapsulating hydrophobic Ce6 and hydrophilic terazamine. Ultrasonic irradiation of ZTC@M NPs activated the Ce6 photosensitizer to produce ROS, further aggravating the hypoxia of the TME and more effectively activating terazamine to kill tumor cells, thus enhancing the chemotherapeutic effect. The new nanomedicine increased the level of ROS to activate the NLRP3 inflammasome (Fig. 3C). During the formation of the NLRP3 inflammasome, caspase-1 was activated, which promoted the transformation of IL-1*β* and IL-18 into mature forms. GSDMD was cleaved by caspase-1, and the release of the GSDMD-N domain caused membrane cleavage and induced pyroptosis. Importantly, the pore-forming ability of GSDMD-N needed to be combined with IL-1*β* secretion to induce pyroptosis. Chen et al.^109^ designed a sonosensitizer, which is composed of a LY364947-loaded porous coordination network (PCN-224) camouflaged with a red blood cell (RBC) membrane. The sonosensitizer generated ROS under US irradiation and initiated pyroptosis *via* the caspase-3/GSDME signaling pathway, causing cell swelling and membrane rupture. In addition, SDT-induced pyroptosis could result in antigen booming with strong maturation of DCs, and LY364947 could facilitate the T-lymphocyte infiltration by depletion of collagen. Furthermore, SDT-induced pyroptotic therapy can stimulate anti-tumor immunological memory to combat tumor recurrence (Fig. 3D).

### 3.4 Mitochondria-targeted therapy

**Figure 4:**
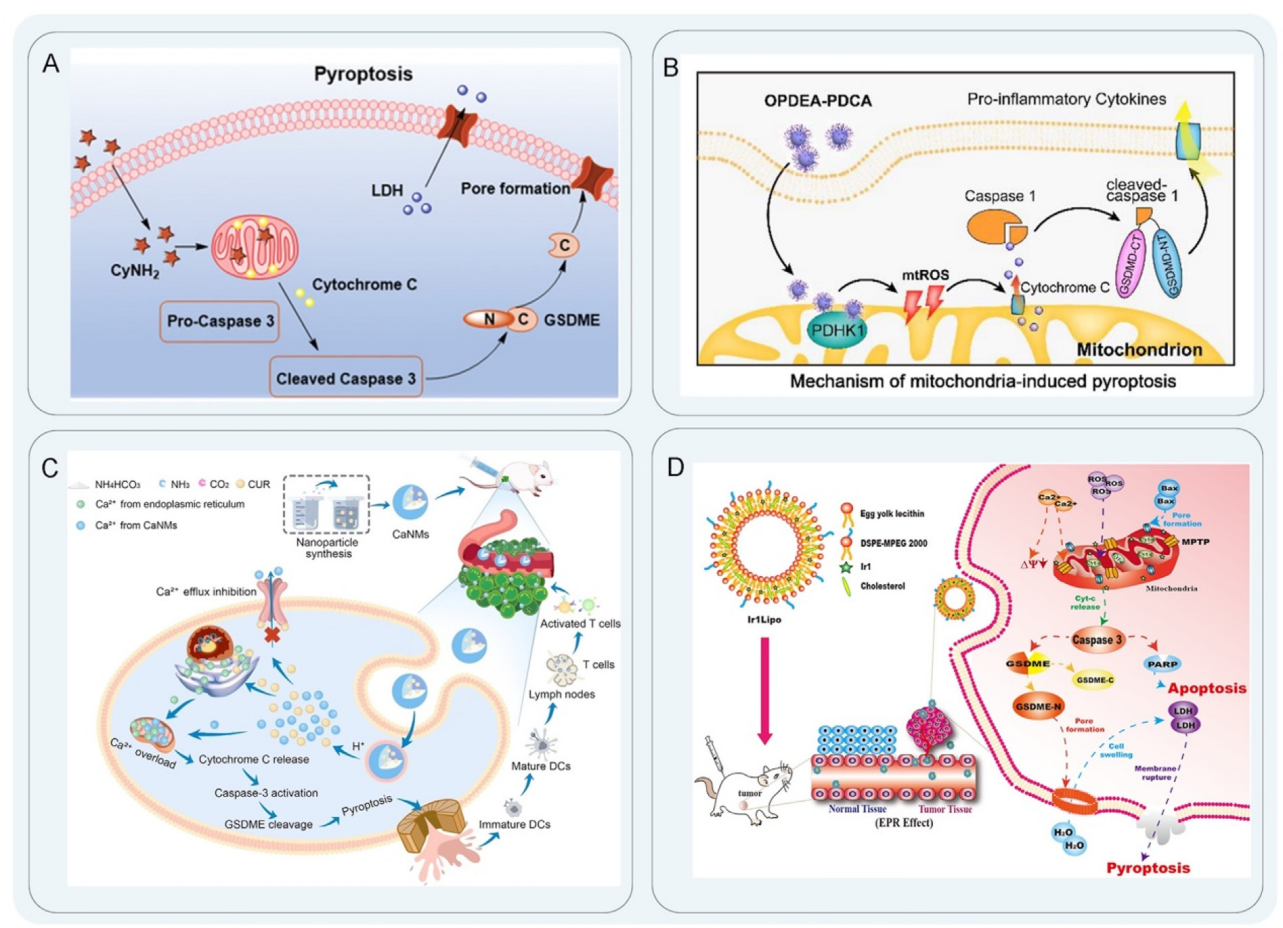
Mitochondria-targeted therapy schematic diagram. (A) Schematic illustration of mechanism map of CyNH_2_-induced pyroptosis. Reprinted with the permission from Ref. 117. Copyright © 2021 Wiley. (B) OPDEA-PDCA initiates mitochondrial oxidative stress and activates caspase-1 to induce pyroptosis. Reprinted with the permission from Ref. 122. Copyright © 2022 American Chemical Society. (C) CaNMs induce pyroptosis by activating caspase-3 to cleave GSDME through mitochondrial Ca^2+^ overload. Reprinted with the permission from Ref. 126. Copyright © 2022 Wiley. (D) The mechanism of Ir1Lipo inducing pyroptosis by increasing ROS level. Reprinted with the permission from Ref. 130. Copyright © 2022 Elsevier.

Mitochondrial-targeted therapy is a therapeutic modality that employs nanomedicines to specifically target mitochondria to induce cell death. In recent years, mitochondria have attracted much attention as a potential target for anti-tumor immunotherapy^111^, ^112^, ^113^. Mitochondria-mediated pyroptosis mainly occurs in the post-apoptotic stage of mitochondria. Mitochondria-mediated apoptosis is induced by ROS, Ca^2+^, and the Bcl-2 protein family^114^ and is accompanied by mitochondrial dysfunction. Activated caspase-3 induces apoptosis, while the tumor suppressor gene GSDME is cleaved by caspase-3 to produce GSDME-N fragments, which perforate the cell membrane to cause pyroptosis^115^^,^^116^. Wang et al.^117^ designed a novel NAD(P)H-quinone oxidoreductase isoenzyme 1 (NQO1)-responsive therapeutic agent (NcyNH_2_) for the treatment of solid tumors. The fluorescence of CyNH_2_ was restored in the presence of NQO1. CyNH2 selectively accumulated in the charged mitochondria, causing mitochondrial membrane damage, leading to the release of cytochrome *c* into the cytoplasm, activating calpain I to cleave GSDME, producing GSDME-N fragments to penetrate the membrane, and inducing pyroptosis to selectively initiate pyroptosis of cancer cells (Fig. 4A). In addition, NcyNH_2_ was encapsulated in a polyethylene glycol (PEG)-poly (lactic*-co-*glycolic acid) block copolymer (PEG-*b*-PLGA) as a nanocarrier for systemic administration, and when combined with the PD-1, it effectively inhibited tumor growth and prolonged the survival time of mice. Mitochondria, as an internal pathway to induce cell death, can change the permeability of their inner and outer membranes. Mitochondria initiate pyroptosis by selectively releasing cytochrome *c*^118^ and other contents into the cytoplasm or using proteases^119^ to control the quality of mitochondria to cause cascade reactions that lead to cell death. Studies have shown that inhibition of pyruvate dehydrogenase kinase 1 (PDHK1) affected the level of mitochondrial quality to increase mitochondrial ROS and decrease the membrane potential to aggravate stress damage in tumors^120^^,^^121^. Jin et al.^122^ synthesized a mitochondria-targeting polymer micelle (OPDEA-PDCA) for selective inhibition of pyruvate dehydrogenase kinase 1 (PDHK1), which could induce pyroptosis by mitochondrial oxidative stress and release immunogenic factors such as IL-1*β* and HMGB1 after cell membrane rupture, leading to a pro-inflammatory TME (Fig. 4B). OPDEA-PDCA could also induce the secretion of soluble PD-L1 molecules. The combined treatment of OPDEA-PDCA and anti-PD-L1 increased the proportion of CD8^+^ T cells and release of IFN-*γ*, which significantly prolongs the activation time of T cells and inhibits the proliferation of osteosarcoma. This study provides a new strategy for mitochondria-targeting pyroptosis to achieve anti-tumor immunotherapy. Ye et al.^123^ designed a macrophage membrane encapsulated in BPTLD (M@BP_TLD_) with mitochondria targeting function to induce pyroptosis as well as immune activation. M@BP_TLD_ could boost the release of immune-activated factors, such as HMGB1 and IL-1*β*, to promote the maturation of DCs. In addition, M@BP_TLD_ and NIR amplified pyroptosis could increase the proportion of M1 type macrophages and CD8^+^ T cells and decrease the proportion of M2 type macrophages, which enhances tumor immunity for TME reprogramming^123^.

A sharp increase of Ca^2+^ in the cytoplasm can lead to mitochondrial damage and irreversible PCD. Through the mitochondrial pathway, Ca^2+^ nanomodulators effectively inhibit tumor proliferation^124^. Under pathological conditions, the dynamic balance between free calcium ions and Ca^2+^ in mitochondria is disrupted. Mitochondria release cytochrome *c* to activate caspase, leading to apoptosis^125^. Therefore, Zheng et al.^126^ designed a Ca^2+^ nanomodulator, CaNMs, composed of CaCO_3_ and curcumin as a pyroptosis inducer for anti-tumor immunotherapy. The release of Ca^2+^ and curcumin at low pH led to a sudden increase in mitochondrial Ca^2+^ ions, which rapidly caused mitochondrial Ca^2+^ overload and increased ROS levels. Subsequently, the activated caspase cleaved GSDME, causing cell swelling and pyroptosis (Fig. 4C). Mitochondrial apoptosis and pyroptosis have a certain crosstalk relationship, and mitochondrial apoptosis promotes the NLRP3 inflammasome assembly^127^^,^^128^. Caspase-3 activates calcium channels and causes calcium efflux, resulting in decreased mitochondrial membrane potential and pyroptosis^129^. In addition, Ca NMs-triggered pyroptosis could promote the maturation of DCs and the activation of CD8^+^ T cells, illustrating the ability to activate a robust immune response. Other researchers have also proposed different nanomedicine designs as new strategies for pyroptosis. Zhang et al.^130^ synthesized and characterized a new ligand, 2-(4′-trifluoromethyl)-[1,1′-biphenyl]-4-yl)-1*H*-imidazo[4,5-*f*][1,10] phenanthroline (TFBIP) and iridium (III) complexes [Ir(ppy)_2_(TFBIP)](PF_6_) (Ir1; ppy is 2-phenylpyridine), [Ir(bzq)_2_(TFBIP)](PF_6_) (Ir2; bzq is benzo[h]quinolone) and [Ir(piq)_2_(TFBIP)](PF_6_) (Ir3; piq is 1-phenylisoquinoline). After encapsulating the complexes into liposomes, Ir1–3 and their corresponding liposomes, Ir1Lipo, Ir2Lipo, and Ir3Lipo, were obtained. Ir1Lipo, Ir2Lipo, and Ir3Lipo increased intracellular ROS levels, causing apoptosis and pyroptosis by reducing mitochondrial membrane potential. The mechanism of cell death was related to mitochondria and was activated by ROS, Ca^2+^, and Bcl-2 protein families. When cytochrome *c* was activated, caspase-3 selectively cleaved poly ADP-ribose polymerase to initiate apoptosis or GSDME to initiate pyroptosis (Fig. 4D).

### 3.5 Chemotherapy

**Figure 5:**
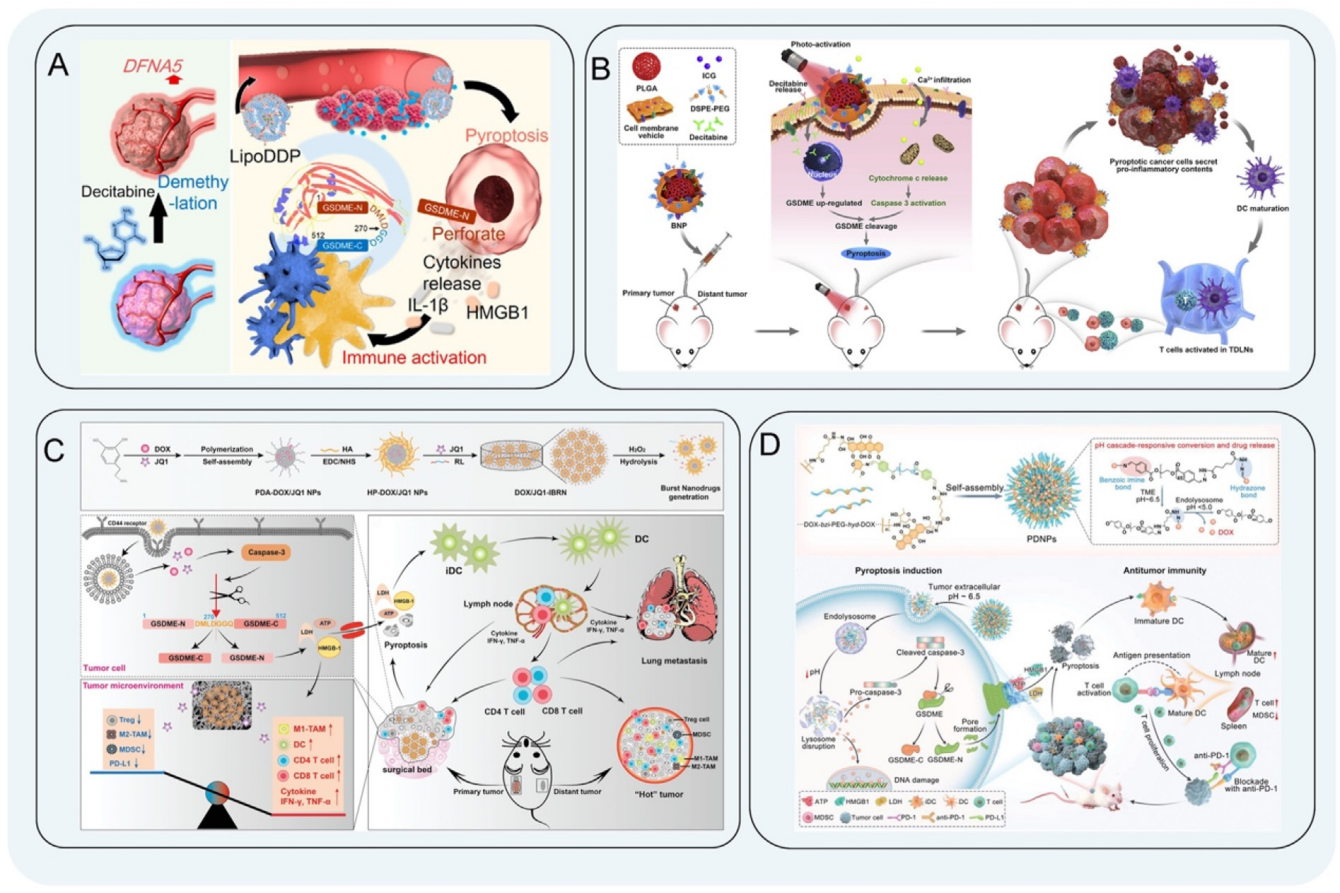
Schematic diagram of mitochondria-targeted therapy. (A) Schematic illustration of mechanism map of LipoDDP triggering pyroptosis to increase the immune effect of chemotherapy. Reprinted with the permission from Ref. 139. Copyright © 2019 American Chemical Society. (B) BNP loaded with indocyanine green and DAC jointly promoted GSDME expression. Reprinted with the permission from Ref. 142. Copyright © 2020 Elsevier. (C) Schematic illustration of the preparation of immunotherapeutic nanoarray DOX/JQ1-IBRN and immune effect. Reprinted with the permission from Ref. 151. Copyright © 2020 Wiley. (D) Schematic illustration of the preparation of PDNP and its release in tumor cells to induce pyroptosis. Reprinted with the permission from Ref. 153. Copyright © 2022 Wiley.

Chemotherapy is a therapeutic modality for tumors that elicits pyroptosis by administering chemotherapeutic agents *via* nanomedicines. A variety of chemotherapeutic drugs have been shown to induce pyroptosis of tumor cells, including cisplatin^131^, paclitaxel^132^, and DAC^133^. Chemotherapy-induced pyroptosis is usually caused by the activation of the GSDME pathway. Because the *DFNA5* gene of the GSDME protein is downregulated in most tumors, DNA demethylating agents can be used to pre-treat the *DFNA5* gene to trigger pyroptosis^134^^,^^135^. Epigenetically modifying drugs that inhibit DNA methylation are mainly divided into two categories: nucleosides and non-nucleosides. Among them, the nucleoside inhibitor DAC is the most clinically used demethylation drug that activates tumor suppressor genes in tumor cells to induce their expression^136^^,^^137^. Differentiating and proliferating cells inhibit DNA synthesis, induce cell death, and increase the killing activity of T cells. Studies have shown that low-dose DAC treatment increases the sensitivity of a variety of cancer cells^138^. Fan et al.^139^ reported tumor-targeted nanoliposomes loaded with cisplatin (LipoDDP) and DAC to trigger pyroptosis. DAC upregulated the expression of GSDME protein, while chemotherapy activated caspase-3/GSDME-mediated pyroptosis with the formation of pores on the cell membrane^140^^,^^141^. Subsequently, many intracellular components, including IL-1*β*, HMGB1, and tumor antigens, were rapidly released. Furthermore, the synergistic effect of DAC and LipoDDP promoted the maturation of DCs and increased the proportion of CTLs within the TME. This novel pyroptosis-based chemotherapy approach not only augments the immunological effects of chemotherapy but also offers valuable insights for tumor immunotherapy (Fig. 5A). For the treatment of primary and metastatic solid tumors, Zhao et al.^142^ developed a biomimetic NP (BNP) loaded with indocyanine green and DAC. BNP-mediated near-infrared (NIR) light activation induced a sharp increase in the cytoplasmic Ca^2+^ concentration and promoted cytochrome *c* release, followed by activation of caspase. By inhibiting DNA methylation, DAC upregulated GSDME expression, triggering caspase-3 cleavage and pyroptosis of cancer cells. There was a notable augmentation in tumor-infiltrating CD45^+^ lymphocytes and mature DCs, an escalation in the proportion of CTLs, and a heightened secretion of IFN-*γ* and TNF-*α*. Ultimately, inflammatory pyroptosis instigated robust maturation of DCs and activated anti-tumor immune responses (Fig. 5B).

The clinical anti-hypertensive drug hydralazine, which acts as a non-nucleoside DNA demethylation agent, has been found to enhance tumor necrosis and impede tumor growth^143^^,^^144^. Furthermore, it exhibits minimal toxicity both *in vivo* and *in vitro*. This, combined with chemotherapy drugs, can expand tumor blood vessels, improve tumor tissue permeability, and enhance the efficiency of nanomedicine delivery. Zhou et al.^145^ prepared multifunctional (M + H)@ZIF/HA NPs by co-encapsulating the chemotherapeutic drug mitoxantrone and DNA demethylating agent hydralazine into a ZIF-8 to counter MDSC-mediated immunosuppression and amplify pyroptosis immunotherapy. Hydralazine upregulated the expression of GSDME, and the released mitoxantrone (MIT) induced the activation of caspase, leading to pyroptosis of tumor cells. In addition, hydralazine prevented the formation of methylglyoxal in MDSCs^146^, thereby triggering a strong cytotoxic T-cell response and significantly eliminating tumors. At the same time, based on the apoptosis-pyroptosis transformation caused by the chemotherapy, the GSDME-N fragment passed through the plasma membrane to trigger pyroptosis, effectively stimulating an anticancer immune response and establishing a long-term immune memory response to fight metastasis. DNA methylation drugs have a certain therapeutic effect in clinical treatment^147^, but a single chemotherapy has a wide range of effects, and the targeting is generally not specific. Therefore, it is necessary to explore new therapeutic methods. Nanomedicines enhance penetration and retention and effectively reduce the side effects of chemotherapy drugs. The synergistic treatment of chemotherapeutic drugs and nanomedicines has broad prospects in future tumor immunotherapy^148^^,^^149^.

Doxorubicin (DOX), also known as adriamycin, is suitable for a variety of malignant tumors in clinical treatment, and it has the advantage of killing proliferating tumor cells. Researchers have improved tumor immunotherapy by preparing multiple modified nanocarriers that are taken up in tumor cells to induce tumor pyroptosis and ICD^150^. Zhao et al.^151^ designed an immunotherapeutic nanoarray called DOX/JQ1-IBRN, where IBRN stands for implantable bio-responsive nanoarray. The combination of DOX and JQ1 significantly triggered GSDME-dependent pyroptosis of tumor cells, further enhancing the number and function of tumor-infiltrating T lymphocytes. JQ1 selectively blocked PD-L1-mediated immune escape, reduced the destruction of Treg, inhibited immune escape and CTL depletion, and then reprogrammed immunosuppressive TME. At the same time, DOX also reduced the expression of PD-1^152^. The two synergistically induced anti-tumor immunity of pyroptosis. In addition, cell surface exposure of the endoplasmic reticulum calcium-binding protein calreticulin and extracellular release of HMGB1 occurred in chemotherapy-induced ICD of tumor cells (Fig. 5C). Liang et al.^153^ developed a supramolecular nanomedicine (PDNP) composed of multiple PEG and DOX drug–polymer hybrid repeats as a pyroptosis inducer. The cascade pH activation of PDNPs achieved deep penetration in tumors. The effective penetration and precise release of chemotherapeutic drugs in tumors effectively induced GSDME-mediated pyroptosis to enhance the immune response (Fig. 5D). In addition, the combination of anti-PD-1 antibodies amplified the anti-tumor effect, promoted the maturation of DCs, initiated CTL proliferation, and inhibited MDSCs.

Melanoma has a high degree of malignancy, and the use of a single-drug treatment method has low efficiency, rapid metastasis, and poor prognosis. Conventional clinical treatment with dabrafenib is suitable for the treatment of metastatic melanoma and inoperable melanoma patients. Celecoxib has anti-tumor effects, such as inhibiting tumor cell proliferation and metastasis, inhibiting tumor angiogenesis, inducing cell differentiation, and regulating immune mechanisms^154^. The non-invasive treatment of two drugs combined with nanomedicines can further induce the efficacy of enhanced immunotherapy for tumor pyroptosis. Zhang et al.^155^ constructed a TME-responsive nanogels (CDNPs) loaded with the BRAF inhibitor (BRAFi) dabrafenib and the cyclooxygenase-2 inhibitor (COX2i) celecoxib, which effectively induced tumor cell pyroptosis. Moreover, CDNPs promoted the maturation of DCs, activated the T cells, and overcame the immunosuppressive function of MDSCs. Meanwhile, CDNPs and *α*PD-1 performed better when used in combination, enhancing the sensitivity of *α*PD-1 and prolonging the survival time of mice^156^.

### 3.6 Photothermal therapy (PTT)

**Figure 6:**
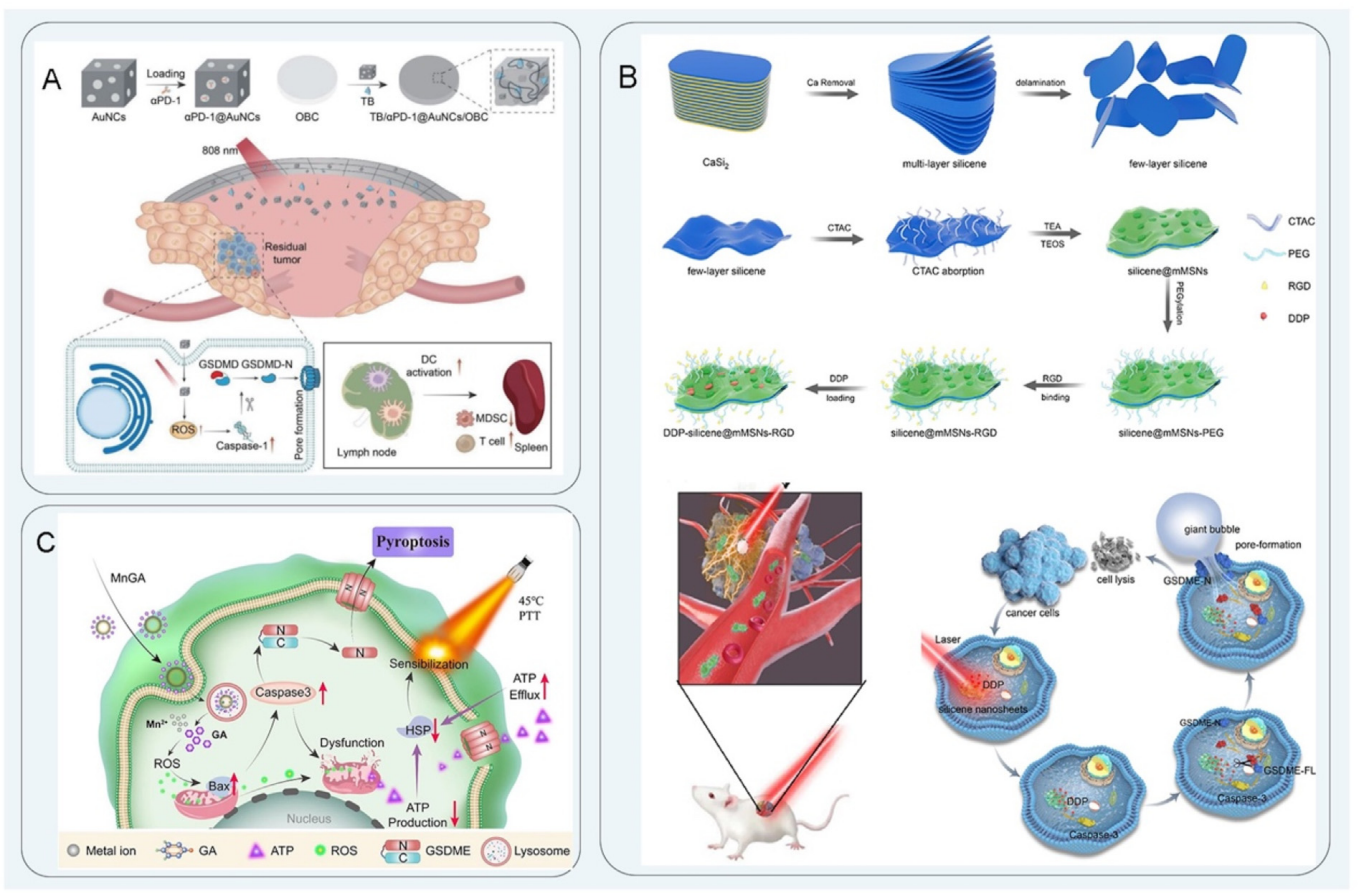
Schematic diagram of PTT. (A) Schematic illustration of *α*PD-1@AuNCs induced pyroptosis, increased ROS levels, and activated caspase to cleave GSDME. Reprinted with the permission from Ref. 164. Copyright © 2022 Tsinghua University Press. (B) The cisplatin-loaded 2D silicene-based nanosystem cleaved the silent GSDME into a cytotoxic GSDME-N terminal, inducing pyroptosis and thermal ablation synergistically. Reprinted with the permission from Ref. 168. Copyright © 2023 Elsevier. (C) Mn-gallate nanoagents effectively produce a large amount of ROS and pyroptosis by triggering caspase by mild PTT. Reprinted with the permission from Ref. 170. Copyright © 2023 Springer Nature.

The fundamental principle of PTT involves the utilization of a photothermal conversion agent, which, when exposed to an external light source, induces the conversion of light energy into heat energy, thereby leading to the direct elimination of tumor cells^157^^,^^158^. PTT can also induce ICD and activate systemic anti-tumor immune responses, including re-distribution and activation of immune effector cells, expression and secretion of cytokines, and transformation of memory T lymphocytes^159^, ^160^, ^161^. Currently, the photothermal materials in PTT include the following: noble metal nanoparticles with a high photothermal conversion efficiency and high unit price; carbon materials with a large photothermal conversion area; several metal and non-metal compounds; and organic dyes^162^. Zhang et al.^163^ designed metal ions (Cu, Fe and Ni) modified to a covalent organic frameworks (COFs) skeleton in a multienzyme-mimicking COFs to reshape the TME. These COFs improved the level of H_2_O_2_ in cells and converted the superoxide radical into H_2_O_2_. It also had excellent PTT performance that accelerated the Fenton-like ionization process to improve the therapeutic effect. Among them, COF-909-Cu strongly induced GSDME-dependent pyroptosis. Under laser irradiation, the expression of GSDME-N was significantly increased, leading to the formation of transmembrane cavities, followed by pyroptosis and the release of many DAMPs. COF-909-Cu promoted the maturation of DCs (CD11^+^ CD103^+^) and significantly increased the proportions of CD4^+^ and CD8^+^ T cells (T_CM_ and T_EM_). In addition, the proportions of MDSCs and Treg cells (CD4^+^ CD25^+^ Foxp3^+^ T cells) were decreased, suggesting that COF-909-Cu-induced pyroptosis effectively reprogrammed the TME and stimulated anti-tumor immunity. Zhou et al.^164^ designed and constructed a novel multifunctional nanomedicine (*α*PD-1@AuNCs) for the treatment of head and neck squamous cell carcinoma. The nanocomposite consisted of oxidized bacterial cellulose, thrombin, and gold nanocages (AuNCs) containing *α*PD-1 antibody, combined with PTT to increase the infiltration of immune cells and control local tumor recurrence. Under NIR irradiation, *α*PD-1@AuNCs showed high photothermal conversion efficiency and induced pyroptosis, increased ROS levels, and activated caspase to cleave GSDME (Fig. 6A). Subsequently, the cell swelling and membrane rupture induced the release of intracellular HMGB1, LDH, ATP, and other contents, which promotes tumor antigen presentation, maturation of DCs, T cell activation for anti-tumor immunity.

In PTT, photothermal materials with good water solubility, strong biosafety, and high photothermal conversion efficiency that reduce thermal damage to normal tissues need to be explored^165^. As an emerging 2D material, silicene has good biodegradability and high photothermal conversion efficiency. Rational design of silicene-based composite materials to broaden their application in tumor therapy is still a key research challenge^166^^,^^167^. Zhang et al.^168^ designed a 2D silicene-based nanosystem loaded with cisplatin, which delivered drugs on demand with high photothermal conversion efficiency. Under NIR laser irradiation, the cisplatin-loaded 2D silicene-based nanosystem quickly generated heat shock, promoted the release of cisplatin to activate caspase, and cleaved the silent GSDME into a cytotoxic GSDME-N terminal, inducing pyroptosis and thermal ablation synergistically, which was of therapeutic significance for clearing melanoma. In addition, LDH is a marker of pyroptosis^169^. Western blotting experiments showed that GSDEM-N expression was significantly upregulated. After DDP-loaded silicene was treated with mMSNs-RGD and NIR laser irradiation, the cell swelling and membrane rupture of tumor cells resulted in the release of a large amount of LDH, which more effectively promoted the anti-tumor activity in the tumor area (Fig. 6B). PTT with a lower treatment temperature can effectively reduce thermal damage of surrounding tissues, which is of great significance for future clinical transformation. Liu et al.^170^ proposed a new strategy for mild PTT by preparing Mn-gallate nanoagents to induce pyroptosis. Mn^2+^ and gallic acid were degraded in an acidic environment, which effectively produced a large amount of ROS to trigger pyroptosis and mitochondrial dysfunction through the caspase-3 pathway, and exhausted ATP, which inhibited the expression of heat shock proteins to achieve mild PTT treatment of osteosarcoma (Fig. 6C). Tao et al.^171^ demonstrated that hollow spheres modified with iron and copper atoms (HCS–FeCu) could induce pyroptosis through ROS–Tom20–Bax–caspase-3–GSDME signaling pathway and enhance anti-tumor immunotherapy. The mild PTT could improve ROS generation and increase the ratio of CD80^+^ CD86^+^ mature DCs, leading to the activation and tumor infiltration of effector T cells, as well as the secretion of INF-*γ*^171^.

### 3.7 Radiotherapy

Radiotherapy is the direct irradiation of the tumor site through high-energy rays-induced DNA damage occurs, but also through the ionizing radiation-induced by the level of ROS in the tumor increased to achieve the effect of tumor treatment. In addition, radiotherapy can induce pyroptosis by promoting CD8^+^ T cell tumor infiltration, thus activating anti-tumor immune effects^172^^,^^173^. Therefore, radiotherapy and immunotherapy are mutually beneficial, and the synergistic effect of radiotherapy and immunotherapy will greatly enhance the efficacy of tumor treatment^174^. Metal radiosensitizing nanomedicines can efficiently absorb radiation energy and increase radiosensitivity to enhance the effect of radiotherapy^175^. Zhou et al^176^. designed a multifunctional nano-enzyme consisting of iodine atoms and ferrocenyl (Fc) groups with catalytic activity as a sensitizer for radiotherapy (TADI–COF–Fc), which can increase X-ray energy deposition to enhance the radiotherapy effect. At the same time, it generates a variety of ROS to inhibit tumor growth^176^. In addition, *in situ* vaccination can also increase radiotherapy. Xu et al.^177^ designed a Fe_3_O_4_ adjuvant (abbreviated as Fe_3_O_4_@Mal/CpG and FMC) for visualization of radiotherapy by magnetic resonance, which was able to activate anti-tumor immunity and increase the therapeutic efficacy of radiotherapy for tumors^177^. Radiotherapy kills tumor cells through various complex mechanisms, including pyroptosis, which can trigger an immune response against the tumor ^178^^,^^179^. Xiao et al.^180^ designed a metal–semiconductor core–shell structure called Au@AgBiS_2_ NPs as a radiosensitizing nanomedicine to inhibit triple-negative breast cancer proliferation and lung metastasis with pyroptosis-induced anti-tumor immunity. When the tumor was irradiated, pyroptosis was induced by activated caspase-1 cleavage of GSDME, which significantly increased the production of ROS, and the levels of TNF-*α* and IFN-*γ* were significantly elevated, promoting the death of tumor cells by T cells and other killer immune cells.

### 3.8 Magnetic hyperthermia therapy (MHT)

MHT is a physical therapy method that enriches magnetic nanomedicine to the tumor site and kills the tumor cells by using the heat-producing effect of the alternating magnetic field^181^. Thermal effect as a key factor for tumor suppression and magnetothermal efficacy, the magnetothermal conversion efficiency of superparamagnetic iron oxide NPs can be improved by the small-size boundary effect of the magnetic NPs themselves and the plasticity of the special structure^182^^,^^183^. Among them, transition metal elements such as Fe, Pt, and Mn are considered to be important elements for the construction of magnetic nanomaterials, which are able to activate the immune system to achieve anti-tumor immunotherapy under the effect of magneto-thermal^184^^,^^185^. Pan et al.^186^ synthesized a superparamagnetic nanomedicine (ZCMF) for the treatment of liver cancer using the strategy of mild MHT, which was able to activate NK cells and inhibit the proliferation of cancer cells significantly under MHT at a controlled temperature of 43–44 °C. MHT has great potential in tumor immunotherapy, which can not only combine with nanomedicines to induce pyroptosis to eliminate tumors but also trigger an anti-tumor immune response and improve the TME to enhance the therapeutic efficacy. Pan et al.^187^ developed a monodisperse, high-performance superparamagnetic CoFe_2_O_4_@MnFe_2_O_4_ synthetic nanomedicines that can effectively thermally ablate primary tumors, generate multiple tumor-associated antigens to promote the maturation and activation of DCs and cytotoxic T cells and trigger systemic immune effects with the therapeutic properties of magnetic materials. Magnetic nanomaterials can be combined with ROS in the TME, and the immune effect is mediated by cellular death^188^. Wang et al.^189^ utilized the dual properties of nano-enzymatic catalysis and magneto-thermal oscillation of platinum-nickel (PtNi) bimetallic “trilobal” nanostructures (PPTNS) to activate caspase-1 and release cytokines, and the levels of TNF-*α* and IFN-*γ* were significantly elevated in tumor tissues to effectively induce cellular death in tumor tissues, which resulted in a significant increase of TNF-*α* and IFN-*γ* levels in tumor tissues. Effectively inducing pyroptosis for tumor immunotherapy.

## 4 Multifunctional composite therapy

**Figure 7:**
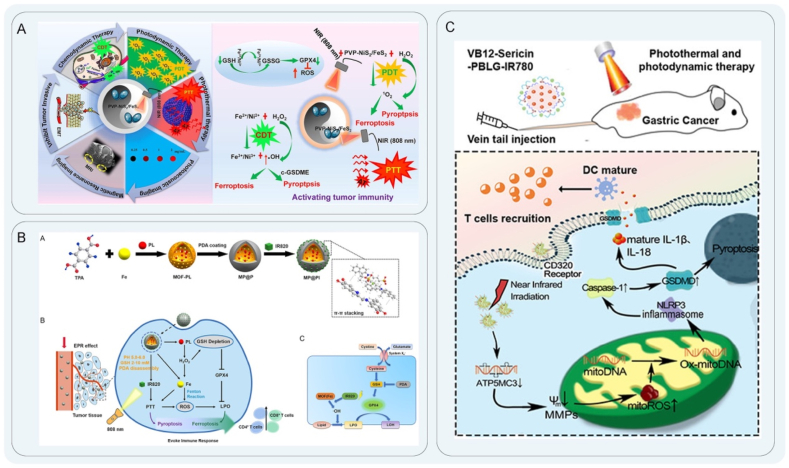
Schematic diagram illustrating mitochondria-targeted therapy. (A) Schematic illustration of PVP-NiS_2_/FeS_2_ synergistic CDT/PDT/PTT therapy activates GSDME to induce pyroptosis for the treatment of metastatic breast cancer. Reprinted with the permission from Ref. 195. Copyright © 2023 Elsevier. (B) MOF NPs were used for PTT and CDT to stimulate cell death. Reprinted with the permission from Ref. 197. Copyright © 2022 American Chemical Society. (C) VB12-sericin-PBLG-IR780 nanomicelles induced pyroptosis by increasing the expression of NLRP3 inflammasome, caspase-1, and GSDMD through PDT/PTT therapy. Reprinted with the permission from Ref. 200. Copyright © 2022 American Chemical Society.

The primary anticancer approach of new tumor immunotherapy is to prompt tumor cell death through a diverse range of treatment modalities. The combination of nanomaterials and multimodal tumor therapy has been found to induce pyroptosis more effectively than either approach alone, resulting in improved tumor inhibition rates and therapeutic efficacy. Numerous scholars have suggested the implementation of multimodal combination therapy as a means of thoroughly eliminating tumor cells and enhancing tumor eradication, thereby reducing the likelihood of tumor recurrence^190^, ^191^, ^192^. Ma et al.^193^ (Table 1) designed a polyprodrug NP, CCNP, composed of a thioether-containing amphiphilic camptothecin as a pyroptosis induction agent. GSDME-mediated pyroptosis was triggered by non-invasive laser irradiation and specific switching of ROS and GSH in the TME. The integration of endogenous and exogenous triggers was used to regulate the path of pyroptosis. Additionally, the utilization of CCNP and NIR treatment has been shown to enhance the recruitment of antigen-processing cells within tumors, as well as promote the maturation and infiltration of DCs. Moreover, the synergistic approach of combining chemotherapy and PDT with CCNP has been found to induce the polarization of macrophages, an increase in CD8^+^ T cells, and a decrease in Treg cells within the TME, suggesting the potential of CCNP in eliciting anti-tumor immune responses. Furthermore, the combination of CCNP with immune checkpoint blockade therapies has demonstrated a significant enhancement in the therapeutic effect, resulting in a systemic immune response and eradication of distant tumors^194^. Xu et al.^195^ prepared a new type of NiS_2_/FeS_2_ NPs modified by polyvinylpyrrolidone (PVP) to prepare PVP-NiS_2_/FeS_2_, which was synergistic with chemodynamic therapy (CDT), PDT, and PTT for the treatment of metastatic breast cancer. Under NIR light irradiation, PVP-NiS_2_/FeS_2_ NPs with photocatalytic activity produced a large amount of ^1^O_2_ and many multivalent ions to trigger ferroptosis and pyroptosis by reducing GSH peroxidase 4 (GPX4) and activating GSDME levels, respectively. In addition, it inhibited metastasis of subcutaneous 4T1 tumors in mice through the epithelial–mesenchymal Transition pathway (Fig. 7A).

**Table 1:** Different nanomedicine for different therapeutic purposes and advantages of nanomedicine induced pyroptosis.

Synthesis of nanoprobes	Size/shape	Pyroptosis pathway	Finding	Therapeutic strategy	Ref.	Advantage of nanomedicine induced pyroptosis
IrP loaded with RG108	198 nm; quasi-spherical	Caspase-3 GSDME	Light-induced pyroptosis and inhibits tumor growth	Photodynamic therapy	68	GSDME protein methyltransferase inhibitor RG108 mediates intracellular GSDME upregulation
PTX-ASC-GO@MP NPs	149.5 ± 3.0 nm	Caspase-3 GSDME	Reducing glucose supply and inhibiting tumor growth	Nanocatalytic therapy	89	Nanomedicine delivery *in vivo* releases ASC plasmid to activate caspase-1
LY364947@PCN-224@membrane (LPM)	90 nm; spherical	Caspase-3; GSDME	Increasing ROS levels and inhibiting tumor growth	Sonodynamic therapy	109	Ultrasound irradiation of nanomedicine generates robust ROS and induces caspase-3 activation
OPDEA-*b*-PDCA	314.5 nm; nanospheres	Caspase-1; GSDMD	Inhibition of osteosarcoma proliferation by mitochondrial oxidative stress	Mitochondria-targeted therapy	122	OPDEA targets mitochondria and PDHK1 causes mitochondrial oxidative stress to induce pyroptosis
(M + H)@ZIF/HA	176.3 ± 1.6 nm; rhombic dodecahedron morphology	Caspase-3; GSDME	Releasing chemotherapeutic drugs and inhibiting tumor growth	Chemotherapy	145	Induction of pyroptosis by delivery of the chemotherapeutic drug (MIT) and DNA demethylating agent hydralazine (HYD)
2D silicene@mMSNs-RGD nanosheets	238 nm; 2D ultrathin silicene nanosheets	Caspase-3; GSDME	Inducing pyroptosis and thermal ablation synergistically	Photothermal therapy	168	NIR activation of 2D silicene core generate heat shock release of DDP-activated caspase-3
Au@AgBiS_2_	94 ± 4.3 nm	Caspase-3; GSDME	Inducing pyroptosis and increasing sensitivity to radiation therapy	Radiotherapy	180	Utilizing the properties of Au, Ag, and Bi elements to efficiently accumulate radiation energy to activate caspase-3 cleavage by GSDME-induced pyroptosis
PPTNS	40.6 ± 7.5 nm	Caspase-1; GSDMD	Magnetic response activates pyroptosis and enhances ROS to impact tumor immunotherapy	Magnetic hyperthermia therapy	189	PtNi bimetallic nanomedicine triggers pyroptosis by magneto-thermal oscillations and nano-enzymatic catalysis
CCNP	53.46 ± 4.09 nm	Caspase-3; GSDME	Triggering a strong immune response and inhibiting tumor growth	Multimodal therapy	193	CCNP is stimulated by the photosensitizer Ce6 and the chemotherapeutic drug CDT acting together in response to ROS/GSH-induced pyroptosis

ASC, apoptosis-associated speck-like protein containing a caspase recruitment domain; ROS, reactive oxygen species; PDHK1, pyruvate dehydrogenase kinase 1; Ce6, chlorin e6; CDT, chemodynamic therapy; GSH, glutathione.

Single-mode PTT cannot completely kill tumor cells, and there are problems with damage to surrounding normal tissues and biosafety of photothermal materials. To solve these limitations of PTT, more research is needed based on PTT combined with other modes of treatment to induce pyroptosis and increase the efficacy of anti-tumor immunity^196^. Deng et al.^197^ designed a polydopamine and photosensitizer IR820-modified MOF nanoplatform for the co-delivery of piperlongumine. The nanosystem was used for PTT and CDT to stimulate cell pyroptosis and ferroptosis. Combining MOF and piperlongumine increased the intracellular H_2_O_2_ concentration and improved the Fenton reaction efficiency. The photosensitizer IR820 induced a photothermal reaction, leading to the activation of caspase-1 and subsequent regulation of GSDMD protein cleavage, resulting in the induction of pyroptosis and the initiation of an immune response *in vivo*. Additionally, the release of intracellular contents, including proinflammatory cytokines (*e.g.*, IL-6, TNF-*α*, and IL-1*β*) and immune stimulants (*e.g.*, HMGB1), occurred during pyroptosis. Moreover, the secretion of HMGB1 was found to exhibit antitumor effects by activating CD4^+^ and CD8^+^ T cells (Fig. 7B). In addition, PTT had a good short-term effect on tumors, while the PDT effects lasted longer. PTT and PDT synergistic therapy complemented each other and solved the drawbacks of both^198^^,^^199^. Guo et al.^200^ prepared VB12-sericin-PBLG-IR780 nanomicelles by coupling sericin derivatives with the tumor-targeting agent VB12 and loading IR780. When PTT was combined with PDT, mitochondrial DNA was damaged, and pyroptosis was induced. *ATP5MC3* was downregulated by the PDT/PTT effect, resulting in decreased mitochondrial membrane potentials, mitochondrial ROS generation, and mitochondrial DNA transformation. Oxidized mitochondrial DNA (ox-mitoDNA) produced by mitochondrial damage increased the expression of the NLRP3 inflammasome^201^, caspase-1, and GSDMD mediated the release of IL-1*β* and IL-18, which led to pyroptosis of gastric cancer cells and further promoted maturation of DCs and recruitment of CD8^+^ and CD4^+^ T cells (Fig. 7C). Sun et al.^202^ reported lysosome-targeted NPs (BDPd NPs) and a pyroptosis-inducing strategy by combined PTT and PDT. The BDPd NPs, when exposed to NIR irradiation, resulted in lysosomal damage and pyroptosis *via* NLRP3/GSDMD and caspase-3/GSDME signaling pathways. This treatment not only effectively suppressed tumor growth and promoted anti-tumor immunity but also enhanced the generation of memory CD4^+^ and CD8^+^ T cells, thereby facilitating the establishment of immune memory. To a certain extent, multimodal therapy can indeed solve the problems faced by single-mode treatment, but the synergistic and therapeutic effects and internal mechanisms of the treatment need to be improved. In the future, more combination strategies will be explored to provide new treatment ideas.

## 5 Conclusions and foresights

Pyroptosis, a recently discovered form of PCD, has been the subject of extensive research. It has been demonstrated that pyroptosis can modulate tumor immunity and elicit a potent inflammatory response, leading to marked tumor regression. This effect is primarily mediated by the gasdermin family, which cleaves cysteine caspases, resulting in immunomodulatory effects. The promotion of tumor cell mutagenesis and acceleration of tumor growth are facilitated by the inflammatory response, whereas the NLRP3 inflammasome regulates immune factors during pyroptosis to sustain homeostasis. A growing body of research indicates a close association between pyroptosis and tumor immunotherapy^203^. With the gradual clarification of the mechanism of the classical and non-classical signaling pathways, more signaling pathways have been gradually discovered. However, the current research on pyroptosis is only a part of the whole picture. The relationship between the pyroptosis mechanism and tumor immunity is complex and needs to be gradually revealed in future research.

Existing anticancer strategies are single-mode methods, such as PDT, chemotherapy, and nanocatalytic therapy, or combined methods, such as multi-mode therapy^204^^,^^205^. These therapeutic strategies can change the redox balance and acid-base balance inside and outside the tumor to effectively inhibit tumor growth and metastasis. To highlight the value of medical and engineering cross-cutting, it is necessary to find precise and targeted tumor treatment strategies promoting intelligent medical treatment. Fortunately, nanomedicine combined with traditional tumor therapy has good development prospects in the field of cancer treatment^206^. The construction of precise functionalized nanomedicine to cope with the complex TME and special tumor diseases has increasingly shown unique advantages, providing a new idea for effectively inducing pyroptosis to treat tumors. Stimulating factors such as ROS and GSH inhibit not only the growth of tumor cells but also the occurrence and development of tumors by changing the conditions of the TME^207^. For the complex tumor environment, it is essential to develop a moderate method to positively induce pyroptosis and maintain the balance of the internal environment. It is necessary to explore the two sides of pyroptosis in tumor immunity and to use nano-preparations to regulate the TME to achieve positive application value.

The design of multifunctional nanomedicine has increasingly shown unique advantages in dealing with complex TME and special tumor diseases. However, the inescapable intricacy of the human body’s milieu presents a formidable challenge. The clinical implementation of nanomedicine remains a distant prospect, and the precise hazards of nanomedicine remain elusive. In the future, we may face challenges, and many key issues remain to be resolved.

I. In terms of nanomedicine, the advancement of precision medicine is poised to investigate novel nanomedicine in conjunction with a wider array of therapeutic modalities. A burgeoning trend in this field is the utilization of nanomedicine for the intravenous administration of drugs, which enables precise targeting of tumors^208^ and tumor imaging^209^. The fundamental prerequisite is to guarantee the safety and stability of high-precision nanomedicine within the human body. For example, inorganic nanomaterials have long residence times in the body and slow metabolism, and their long-term use may cause harmful toxic accumulation; biological nanomaterials have the disadvantages of rapid metabolism and instability, so it is necessary to avoid the risk of being cleared by the immune system after recognition. Researchers should try to explore the versatility of nanomedicine and consider the optimization of its physical and chemical properties. The research direction is to improve the accuracy of drug targeting and *in vivo* circulation stability and promote the effectiveness between nanomedicine and immunotherapy.
It is imperative to address concerns regarding the side effects and potential risks associated with the *in vivo* use of nanomedicines. There are significant differences in the pharmacokinetics of nanomedicines of different nano-sizes, and the development of nanomedicines to improve their safety in the future should be considered in the process of preparation from the toxicological aspects, pharmacological aspects, and data from clinical practice^210^^,^^211^. First, the component detection of nanomedicines needs to be systematically analyzed *in vitro* and *in vivo*^212^. Changes in morphology in the characterization of nanomedicines may have an impact on the release and stability of the drug, such as particles, micelles, liposomes, and other types of nanomedicines or polycrystalline materials^213^. To ensure the feasibility of nanomedicine, strict quality control should be carried out according to the preparation standards^214^. Secondly, the degradation factor of nanomedicine is also one of the potential risks; *in vitro,* it will be affected by environmental factors, and agglomeration, degradation, and depletion may occur, and the necessary *in vitro* stability test can solve the problem. The release, uptake, and enrichment of nanomedicines *in vivo* is complex, and trying to make nanomedicines reach the tumor site is a major focus in research^215^. The uptake concentration of nanomedicine is not as high as that of injection, and larger particle sizes may be retained in organs such as lungs, liver, kidneys, etc. and even some small molecules of nanomedicine may pass through the biological barrier. For the metabolism, the half-life time and clearance time of nanomedicines can be regulated to reach within the safety range of nanomedicines^216^. Finally, since the existing studies in animal models do not fully predict the actual physiological and pathological responses in the human body, detailed physicochemical characterization *in vitro* and *in vivo,* as well as clinical safety assessment, can only reduce the risk of nanomedicines *in vivo* applications. Therefore, achieving the absolute safety of nanomedicines in clinical use is a great challenge.
II. In terms of pyroptosis, the molecular mechanism of pyroptosis has not been studied in depth, and there may be more signaling pathways related to pyroptosis that remain to be explored ^217^. Gasdermin protein is a key factor in inducing pyroptosis, and many members of the gasdermin family have not been defined. In addition, the cleavage of GSDM protein is not limited to the caspase pathway, and there are other protease cleavage sites involved in the process of pyroptosis. Because different PCD pathways can be transformed into each other, more pathways of pyroptosis can be explored in the future to provide new strategies for anti-tumor immunotherapy.
III. In terms of immunity, firstly, nanomedicine can achieve anti-tumor immunity by inducing tumor cell death and achieving anti-tumor immunity^218^. Most nanomedicine are cleared by the immune system when they enter the body and are difficult to intake and express at the tumor site, so it is important to ensure that nanomedicine is not cleared and at the same time stimulate anti-tumor immunity to take advantage of the nanomedicine’s performance^219^. Nanomedicine has great application value and development prospects in inducing anti-tumor immunity. Second, cell death can regulate the homeostasis of the immune system, and the relationship between different modes of cell death and immunity is intricate. There are more unknown puzzles to be explored regarding the effect of the cell death process on tumor immunity^220^. Finally, novel single-modality tumor therapies can stimulate anti-tumor immune effects and inhibit tumor growth, metastasis, and recurrence, which show greater potential in tumor therapy. In recent years, many immune targets and nanomedicine for tumor immunotherapy have emerged, and combining different novel therapeutic modalities in future research will bring discoveries in tumor therapy. In addition, Multimodal treatment approaches offer the benefit of activating the immune system from various angles, thereby significantly augmenting the effectiveness of anti-tumor immunity and accomplishing the objective of eliminating malignant cells^221^. This therapeutic modality entails the coordinated administration of multiple pharmacological agents, which reinforces the body’s therapeutic response to a certain degree. The tolerance and side effects should be considered to avoid increasing the damage to the surrounding normal tissues. Moreover, multimodal treatment is a relatively new treatment strategy, which may involve more significant risks that need to be considered carefully.

**Scheme 3:**
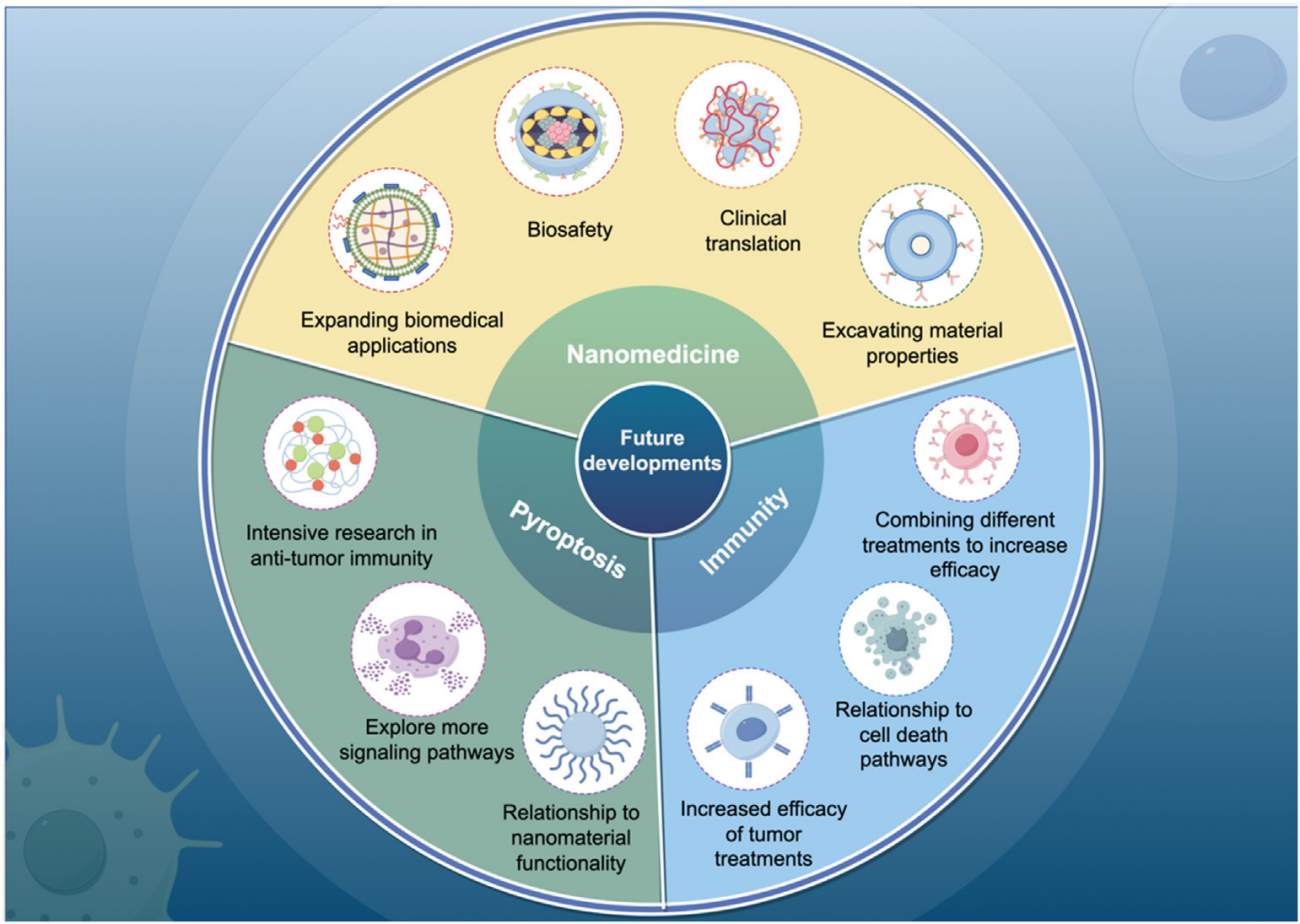
Schematic illustration of the direction of development of nanomedicine in the future.

With the ongoing expansion and advancement of nanomaterial research, the field has demonstrated exceptionally vast potential in the realm of tumor treatment. Leveraging its distinctive characteristics and attributes, nanomedicine is anticipated to propel a significant advancement in tumor therapy in the foreseeable future. We look forward to bringing more significant benefits to tumor therapy in the future (Scheme 3).

## Author contributions

Dengbin Wang and Defan Yao conceived the project and supervised the project. Yuelin Huang searched the references and wrote the manuscript. Chunting Wang and Yanhong Chen proofread and polished the manuscript. All of the authors have read and approved the final manuscript.

## Conflicts of interest

The authors have declared that no conflict of interest exists.
